# Bitter Peptides YFYPEL, VAPFPEVF, and YQEPVLGPVRGPFPIIV, Released during Gastric Digestion of Casein, Stimulate Mechanisms of Gastric Acid Secretion *via* Bitter Taste Receptors TAS2R16 and TAS2R38

**DOI:** 10.1021/acs.jafc.2c05228

**Published:** 2022-09-02

**Authors:** Phil Richter, Karin Sebald, Konrad Fischer, Maik Behrens, Angelika Schnieke, Veronika Somoza

**Affiliations:** † Leibniz Institute for Food Systems Biology at the Technical University of Munich, Lise-M eitner-Straße 34, 85354Freising, Germany; ‡ Chair of Livestock Biotechnology, TUM School of Life Sciences, Technical University of Munich, Liesel-Beckmann-Straße 1, 85354Freising, Germany; § Chair of Nutritional Systems Biology, TUM School of Life Sciences, Technical University of Munich, Lise-Meitner-Straße 34, 85354Freising, Germany; ∥ Department of Physiological Chemistry, Faculty of Chemistry, University of Vienna, Josef-Holaubek-Platz 2 (UZA II), 1090Wien, Austria

**Keywords:** casein, bitter peptides, gastric acid secretion, bitter taste receptors, HGT-1, gastric digestion

## Abstract

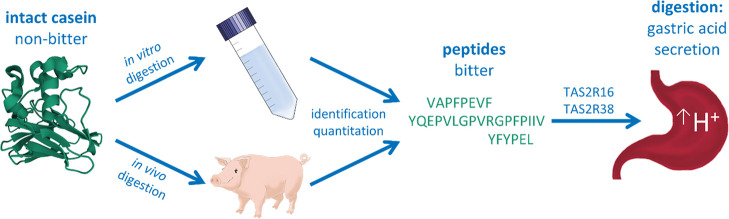

Eating satiating, protein-rich foods is one of the key aspects of modern diet, although a bitter off-taste often limits the application of some proteins and protein hydrolysates, especially in processed foods. Previous studies of our group demonstrated that bitter-tasting food constituents, such as caffeine, stimulate mechanisms of gastric acid secretion as a signal of gastric satiation and a key process of gastric protein digestion *via* activation of bitter taste receptors (TAS2Rs). Here, we tried to elucidate whether dietary non-bitter-tasting casein is intra-gastrically degraded into bitter peptides that stimulate mechanisms of gastric acid secretion in physiologically achievable concentrations. An *in vitro* model of gastric digestion was verified by casein-fed pigs, and the peptides resulting from gastric digestion were identified by liquid chromatography–time-of-flight-mass spectrometry. The bitterness of five selected casein-derived peptides was validated by sensory analyses and by an *in vitro* screening approach based on human gastric parietal cells (HGT-1). For three of these peptides (YFYPEL, VAPFPEVF, and YQEPVLGPVRGPFPIIV), an upregulation of gene expression of *TAS2R16* and *TAS2R38* was observed. The functional involvement of these TAS2Rs was verified by siRNA knock-down (kd) experiments in HGT-1 cells. This resulted in a reduction of the mean proton secretion promoted by the peptides by up to 86.3 ± 9.9% for TAS2R16kd (*p* < 0.0001) cells and by up to 62.8 ± 7.0% for TAS2R38kd (*p* < 0.0001) cells compared with mock-transfected cells.

## 1 Introduction

The consumption of protein-rich foods and the reduction of fats and carbohydrates are high on the priority list in modern diets. Numerous studies have shown that increased protein intake can reduce food intake and, consequently, the body fat mass and body weight.^1^ One of the key studies from the group of Westerterp-Plantenga^2^ shows that a higher casein content in the diet of healthy subjects (10% *vs* 25% of energy) leads to an increased feeling of fullness and satiety. Mechanistically, dietary protein has been shown to stimulate the release of hormones in the intestine, such as glucagon-like peptide 1 (GLP-1), cholecystokinin, and peptide YY, which promote the feeling of satiation.^3^ For example, the plasma concentration of GLP-1, which is released in the ileum and colon, increased after administration of a high-protein breakfast (60% protein) compared to a high-fat or high-carbohydrate breakfast.^4^

However, mechanisms of satiation are not only initiated during digestion in the intestines but also already in the stomach. Here, food ingredients regulate gastric motility and emptying as well as gastric acid secretion.^5^ Dietary satiating effects have been demonstrated not only for complex proteins but also for a number of their constituents, namely, peptides and amino acids.^6^

Besides the effects on hormones promoting satiation, a reduction of food intake by dietary proteins can also be achieved by regulation of the so-called “hunger hormone” ghrelin, which promotes the feeling of hunger.^7^ In a previous study by Blom *et al.*,^8^ mean gastric ghrelin release was reduced by 46% after intake of a high-protein diet (58.1% of energy from protein and 14.1% of energy from carbohydrate) as compared to a high-carbohydrate diet (19.3% of energy from protein and 47.3% of energy from carbohydrate). A similar involvement of the stomach in the regulation of food intake was shown by Uchida *et al.*,^9^ where administration of a dose of 1 g per kg body weight of the bitter-tasting amino acid l-arginine to male Sprague–Dawley rats resulted in slowing of gastric emptying. In one of our own previous studies, l-arginine also promoted slowing of gastric emptying and a decrease in energy intake in healthy subjects.^10^ In addition, ingestion of l-arginine-enriched wheat protein hydrolysate increased plasma concentrations of the satiating neurotransmitter serotonin.^10^

For l-arginine, one of the most bitter-tasting amino acids in our diet, our group also demonstrated a stimulation of cellular mechanisms regulating gastric acid secretion in cultured human parietal cells (HGT-1) *via* TAS2R1 signaling.^11,12^ The underlying hypothesis of bitter-taste-sensing chemoreceptors being involved in gastric acid secretion was verified by preceding experiments, showing that the bitter-tasting caffeine stimulates (i) proton secretion *via* TAS2R signaling in TAS2R43 CRISPR-Cas9-edited human parietal HGT-1 cells in culture and (ii) promotes gastric acid secretion in healthy subjects, which was reduced by co-administration of the TAS2R antagonist homoeriodictyol.^13^ Notably, administration of bitter-masking homoeriodictyol not only reduced the caffeine-evoked effect on gastric acid secretion but also increased gastric motility and emptying, decreased peripheral serotonin levels, and stimulated appetite.^14^

From a physiological perspective, the stomach is able to sense peptides and amino acids, which then regulates the release of hormones gastrin and motilin, stimulating gastric acid secretion as well as gastric motility and emptying.^15^ Motilin receptors are activated not only by the hormone itself but also by agonists such as the bitter-tasting drug erythromycin, which activates TAS2R10^16^ or denatonium benzoate, targeting various TAS2Rs. In addition to amino acids,^11^ peptides present in casein hydrolysates are well-known for their bitter taste.^17^ Already seven decades ago, the bitter taste of dairy products was ascribed to casein peptides generated upon casein hydrolysis.^18^ The molecular mechanisms underlying the bitter taste of casein peptides have been elucidated more recently, that is, by Maehashi *et al.*(19) who demonstrated that casein hydrolysates activate TAS2R16 in transfected HEK293 cells. Since then, several peptides have been demonstrated to activate a number of bitter receptors, namely TAS2R1, TAS2R4, TAS2R14, TAS2R39, and TAS2R46,^20^ although no specific peptide sequences are known to chiefly result in TAS2R16 activation, and peptides do not conform with the so far identified selectivity of TAS2R16 for β-d-glucopyranosides.

Activation of the G-protein-coupled TAS2Rs in gastric parietal cells is based on binding of taste-active compounds, resulting in increased enzymatic activity of phospholipase C β2.^13^ In some cases, the presence of agonists in the nanomolar range is sufficient to activate TAS2Rs.^21,22^ The product resulting from the phospholipase C β2 activity, phosphatidylinositol-4,5-bisphosphate (PIP_2_), is cleaved into diacylglycerol and inositol trisphosphate (IP_3_), which leads to calcium release from the endoplasmic reticulum.^23^ The increased calcium concentration in the cell promotes the activity of H^+^,K^+^-ATPase, which transports protons out of the parietal cell by cleaving ATP.^24^ Similarly, activation of the G-protein-coupled receptors H_2_ by histamine and M_3_ by acetylcholine increases proton secretion in gastric parietal cells. Binding of acetylcholine also causes activation of phospholipase C β2, and receptor H_2_ activates adenylyl cyclase, which catalyzes the formation of cAMP.^25^ For the proposed mechanism of proton secretion induced by bitter compounds in parietal cells, see Supporting Information Figure-SI 1.

Dietary intake of bitter compounds is recognized by TAS2Rs located on taste cells of the tongue’s taste buds. However, structural changes of food constituents during gastric digestion may also lead to the formation of compounds with bitter taste quality, which are not sensed as bitter tasting due to the lack of appropriate nerve connections between the stomach and the brain. For example, tryptic digestion of bovine casein releases peptides that have a bitter taste, whereas the intact protein does not taste bitter.^26^ The formation of peptides in the stomach is catalyzed by the gastric enzyme pepsin.^27^ Its inactive precursor pepsinogen is autocatalytically cleaved into the active form pepsin at pH values below 6.^28^ Pepsin preferably cleaves next to the amino acids phenylalanine, tyrosine, and leucine, but it is able to hydrolyze almost all peptide bonds.^29^ At pH 7 and higher, the enzyme denatures irreversibly.

In this work, we hypothesize (i) that bitter peptides are formed during gastric digestion of non-bitter-tasting bovine casein, and (ii) that these bitter peptides have an effect on mechanisms regulating gastric acid secretion *via* TAS2Rs. Verification of this hypothesis could foster research on taste qualities of dietary proteins and their potential as food constituents that help to modulate food intake and, ultimately, maintain a healthy body weight.

## 2 Materials and Methods

### 2.1 Chemicals

1,5-Carboxy-seminaphtorhodafluor acetoxymethylester (SNARF-1-AM) and Dulbecco’s modified Eagle’s medium GlutaMAX (DMEM) were purchased from Thermo Fisher Scientific. Fetal bovine serum (FBS Supreme), trypsin/ethylenediaminetetraacetic acid, and penicillin–streptomycin were obtained from PAN-Biotech GmbH (Aidenbach, Germany). Phosphate buffered saline was bought from Biozym Scientific GmbH (Hessisch Oldendorf, Germany). Dimethyl sulfoxide (DMSO) was purchased from Carl Roth (Karlsruhe, Germany). CaCl_2_, casein from bovine milk, 3-(4,5-dimethylthiazol-2-yl)-2,5-diphenyltetrazolium bromide (MTT), d-glucose, formic acid, HCl, 4-(2-hydroxyethyl)-1-piperazineethanesulfonic acid (HEPES), KCl, KH_2_PO_4_, KOH, MeCN, MgCl_2_(H_2_O)_6_, MgSO_4_, NaCl, NaHCO_3_, (NH_4_)_2_CO_3_, and pepsin from porcine gastric mucosa were ordered from Merck KGaA (Darmstadt, Germany). Custom peptides (PVVVPPFLQPEVM, VAPFPEVF, YFYPEL, YQEPVLGPVRGPFPIIV, and YYVPLGTQ) were synthesized by Genscript Biotech with a purity of >95% (New Jersey, USA). Double-distilled water (ddH_2_O) from Elga Purelab Classic (Veolia Water Solutions & Technologies, France) was used for all experiments. Krebs-Ringer–HEPES buffer (KRHB) contains 130 mM NaCl, 4.7 mM KCl, 1.3 mM CaCl_2_, 1.2 mM MgSO_4_, 1.2 mM KH_2_PO_4,_ 11.7 mM d-glucose, and 10 mM HEPES; the pH was adjusted to 7.4 with KOH.

### 2.2 *In Vitro* Digestion

The *in vitro* digestion was based on the Nature protocol established by Brodkorb *et al.*(30) For this, simulated salivary fluid (SSF, containing 15.1 mM KCl, 3.7 mM KH_2_PO_4_, 13.6 mM NaHCO_3_, 0.15 mM MgCl_2_(H_2_O)_6_, 0.06 mM (NH_4_)_2_CO_3_, 1.1 mM HCl, and 1.5 mM CaCl_2_(H_2_O)_2_) and simulated gastric fluid (SGF, containing 6.9 mM KCl, 0.9 mM KH_2_PO_4_, 25.0 mM NaHCO_3_, 47.2 mM NaCl, 0.12 mM MgCl_2_(H_2_O)_6_, 0.5 mM (NH_4_)_2_CO_3_, 15.6 mM HCl, and 0.15 mM CaCl_2_(H_2_O)_2_) were prepared. A total amount of 100 mg casein was suspended in 3 mL of SSF and incubated for 5 min at 37 °C in the tube rotator. After taking a sample (500 μL, *t* = 0 h), 2 mL of SGF was added and the pH was adjusted to 3 with 100 μL of HCl (1 M). After addition of 125 μL of pepsin solution (80,000 U/mL in 10 mM HCl), 275 μL of H_2_O was added to fill up to 5 mL. This was followed by further incubation at 37 °C in a tube rotator for 6 h. 500 μL of samples was taken at 0.25, 0.5, 0.75, 1, 2, 3, 4, 5, and 6 h, respectively. The samples were frozen in liquid nitrogen and stored at −80 °C until further analysis. For MS experiments, all samples were diluted 1:1 with 10% MeCN. To prepare the casein hydrolysate after 1 h of digestion for use in cell assays, the samples were desalted (20 mL H_2_O + 0.1% formic acid) by solid phase extraction (HyperSep C18, 5 g, Thermo Scientific) and peptide fraction was then eluted with 20 mL each of 20% MeCN + 0.1% formic acid and 60% MeCN + 0.1% formic acid.^31^

### 2.3 *In Vivo* Digestion

For *in vivo* experiments, 1 g of casein was suspended in 5 mL of H_2_O and fed to pigs (German Landrace, German Landrace × minipig, age: 16–27 weeks). After 2 h, the pigs were anesthetized and killed and the stomach was removed. The stomach contents were aliquoted, frozen in liquid nitrogen, and stored at −80 °C until further analysis. To remove any impurities, the samples were desalted (1 mL H_2_O + 0.1% formic acid) by solid phase extraction (Discovery DSC-18, 100 mg, Sigma-Aldrich) and the peptide fraction was then eluted with 750 μL each of 20% MeCN + 0.1% formic acid and 60% MeCN + 0.1% formic acid. After solvent removal, the peptides were dissolved in 5% MeCN.

### 2.4 Ultra-performance Liquid Chromatography–Time-of-Flight Mass Spectrometry and Peptide Identification

Measurements were performed using a Sciex ExionLC AC (Sciex, Darmstadt, Germany) coupled to a Sciex TripleTOF 6600 mass spectrometer (Sciex, Darmstadt, Germany). Data acquisition and instrumentation control were performed with AnalystTF software (v 1.7.1; Sciex, Darmstadt, Germany). Separation was performed using a 100 × 2.1 mm, 1.7 μm, Kinetex C18 column (Phenomenex, Aschaffenburg, Germany) with a guard column of the same type with 0.1% aqueous formic acid and acetonitrile containing 0.1% formic acid at a flow rate of 0.3 mL/min. The gradient was based on the following scheme: 0 min, 5% B; 0.5 min, 5% B; 14 min, 40% B; 15 min, 98% B; 16 min, 98% B; 17 min, 5% B; and 20 min, 5% B. The column oven temperature was set at 40 °C, and the injection volume was 1 μL per sample. For ToF-MS measurements, the same parameters^32,33^ were used for all samples (ion spray voltage 5500 eV, source temperature 550 °C, nebulizing gas 55 psi, and heating gas 65 psi). Nitrogen was set to 35 psi and served as a curtain gas to effectively dissolve the ions. In IDA mode, a ToF-MS survey scan was acquired from *m*/*z* 100 to 1500 using an accumulation time of 250 ms (declustering potential DP 80 V and collision energy CE 10 V). Product ion spectra for the 15 most abundant compounds in the *m*/*z* range of 100–1500 were recorded in high sensitivity mode for 50 ms (DP 80 V, CE 45 V, CE spread *CES* 15 V). MaxQuant software (v 1.6.3.4; Max Planck Institute of Biochemistry, Planegg, Germany) compares the data found in the recorded MS/MS spectra with in silico-generated spectra.^34^ With the selected settings (peptide length between 4 and 25 amino acids, unspecific digestion, variable modifications: oxidation M, acetyl protein N-term, carbamidomethyl C, phospho STY, andromeda score > 10) and imported sequences of α_S1_-casein (UniProt P02662), α_S2_-casein (UniProt P02663), β_A1_-casein (UniProt P02666, natural variant A1), β_A2_-casein (UniProt P02666, natural variant A2), and κ-casein (UniProt P02668), which have lengths between 190 and 224 amino acids, ≈12,000 different peptides are possible. The specific cleavage pattern of pepsin limits the number of peptides that can be generated. However, in order not to exclude the pepsin-independent formation of peptides caused by gastric acid, an enzyme-independent in silico digest with all possible ≈12,000 peptide spectra was chosen.

### 2.5 Quantitative ^1^H Nuclear Magnetic Resonance Spectroscopy

The synthesized reference peptides were dissolved in D_2_O. 600 μL of each of the peptide solutions was loaded into NMR tubes (178 × 5 mm inner diameter, USC tubes, Bruker, Rheinstetten, Germany) and analyzed using a 400 MHz Avance III NMR spectrometer (Bruker, Rheinstetten, Germany). Instrument calibration and data processing were performed as detailed earlier.^35^ The specific proton resonance signal at 3.55 ppm (s, 3H) of the external standard caffeine (3.58 mmol/L) was used for calibration. The calibration was verified immediately before the measurement with l-tyrosine (4.34 mmol/L, 7.10 ppm, d, 2H).

### 2.6 Targeted Proteomics

All targeted proteomics LC–MS/MS measurements were performed using a Sciex ExionLC AC (Sciex, Darmstadt, Germany) coupled to a 6500+ QTrap LC–MS/MS system (Sciex, Darmstadt, Germany) operating in the positive electrospray ionization mode. Data acquisition and instrumentation control were performed with AnalystTF software (v 1.7.1; Sciex, Darmstadt, Germany). Separation was performed using a 100 × 2.1 mm, 1.7 μm, Kinetex C18 column (Phenomenex, Aschaffenburg, Germany) with a guard column of the same type with 0.1% aqueous formic acid and acetonitrile containing 0.1% formic acid at a flow rate of 0.25 mL/min. The gradient was based on the following scheme: 0 min, 15% B; 7 min, 40% B; 7.5 min, 98% B; 10.5 min, 98% B; 10.8 min, 15% B; and 15 min, 15% B. The column oven temperature was set at 40 °C, and the injection volume was 1 μL per sample. For MS/MS measurements, the same parameters were used for all samples (ion spray voltage 5500 eV, source temperature 450 °C, nebulizing gas 60 psi, and heating gas 30 psi). Nitrogen was set to 35 psi and served as a curtain gas to effectively dissolve the ions. To optimize the parameters DP, CE, and collision cell exit potential for each peptide and transition (Supporting Information Table-SI 2), standard solutions of the five synthesized peptides were injected directly into the MS ion source. Ionization parameters were optimized in positive ESI mode using AnalystTF software (v 1.7.1; Sciex, Darmstadt, Germany). For the preparation of the calibration curves, five peptides were dissolved individually in D_2_O (600 μL) and the concentration of the stock solutions was determined by qNMR.^35^ For each peptide, seven calibration solutions (0.5, 1, 5, 10, 50, 100, and 200 μM) were diluted in 5% MeCN from these stock solutions. The data were analyzed using MultiQuant software (v 3.0.3; Sciex, Darmstadt, Germany).

### 2.7 Sensory Study

To verify the bitterness of the selected peptides, each peptide was dissolved in bottled, non-carbonated water (1.5 mM) and tested by 17 panelists in a three-alternative forced choice (3-AFC) test against the bottled, non-carbonated water.^36^ To prevent ingestion of toxic substances, the purity of the peptides was checked (>95%, LC–MS) and the solutions were spit out and not swallowed.

### 2.8 Cell Culture

Human gastric tumor cells (HGT-1), provided by Dr. C. Laboisse, Nantes (France), were cultivated at 37 °C in a humidified atmosphere at 5% CO_2_ in DMEM containing 10% FBS and 1% penicillin and streptomycin. Cells between passages 15 and 29 were used for all experiments. 50,000 cells per well were seeded 1 day before the experiment into a transparent 96-well plate, for cell viability assays, or into a black 96-well plate, for proton secretion assays. For the detection of gene expression, 800,000 cells per well were seeded into a T25 cell culture flask.

### 2.9 Cell Viability

To exclude cytotoxic effects of all used substances on HGT-1 cells, their metabolic activity was tested using MTT dye. For this purpose, cells were treated with solutions of casein (10 μM), hydrolysates (10 μM), peptides (250 μM), and probenecid (1 mM) in KRHB or the transfection reagents for either 30 min or 72 h under standard conditions. The solutions were removed, and 100 μL of MTT solution (0.83 mg/mL in DMEM) was added to each well. After another incubation for 15 min under standard conditions, the MTT solution was removed, and the formed formazan was dissolved in DMSO. Absorbance was measured at 570 nm (reference 650 nm) using an Infinite M200 plate reader (Tecan, Switzerland). Cell viability was calculated relative to cells treated with KRHB only (=100%).

### 2.10 Proton Secretion Assay

The measurement of proton secretion from HGT-1 cells represents a well-established model for the identification of bitter compounds. By affecting extraoral bitter taste receptors with bitter compounds, a modulatory effect on proton secretion can be measured. For this purpose, cells were washed with KRHB and incubated with 3 μM of the intracellular pH indicator 1,5 carboxy-seminaphto-rhodafluor acetoxymethyl ester (SNARF-1-AM) under standard conditions. After 30 min, the cells were washed again with KRHB and then incubated with casein (0.01–10 μM equimolar related to the relevant forms α_S1_- and β-casein), hydrolysate (0.01–10 μM), or the peptides (0.01–200 μM). For co-incubation experiments, peptides were incubated together with probenecid (1 mM). All substances were dissolved and diluted in KRHB. 1 mM histamine was used as a positive control. Measurements were performed using FlexStation 3 (Molecular Devices, USA). The excitation wavelength was 488 nm, and the emission wavelengths were 580 and 640 nm. For calibration (pH range 7.0–8.0), the intracellular and extracellular pH was adjusted with 2 μM nigericin in potassium buffer (20 mM NaCl, 110 mM KCl, 1 mM CaCl_2_, 1 mM MgSO_4_, 18 mM d-glucose, and 20 mM HEPES). The intracellular proton index (IPX) was calculated as the log2 value of the 580/640 ratio and compared with cells without treatment. Negative values represent increased secretion of protons and therefore stimulation of mechanisms regulating gastric acid secretion in HGT-1 cells. In contrast, positive values represent an inhibition of secretion compared to the untreated control.

### 2.11 Quantitation of mRNA Expression

For mRNA expression analysis, 800,000 cells were seeded in a T25 cell culture flask (25 cm^2^) 1 day before the experiment. After incubation with 17.5 μM VAPFPEVF, 0.03 μM YFYPEL, or 0.4 μM YQEPVLGPVRGPFPIIV for 15, 30, 60, and 120 min, RNA was isolated using the peqGOLD RNA Kit (VWR Peqlab, USA) following the manufacturer’s protocol. Determination of RNA concentration (A260/A280 between 2.03 and 2.09) was performed on a NanoDrop One^c^ (Thermo Fisher Scientific Inc., USA). RNA integrity number (RIN 9.9–10.0, version 2.6, assay class Eukaryote Total RNA Nano) was analyzed using a 2100 Bioanalyzer (Agilent Technologies, USA). Removal of gDNA and synthesis of cDNA were performed using the iScript gDNA Clear cDNA Synthesis Kit (BioRad, Feldkirchen, Germany) following the manufacturer’s protocol. Real-time-qPCR (RT-qPCR) was performed with 50 ng cDNA amplified with SsoAdvanced Universal SYBR Green Supermix (Bio-Rad Laboratories, Inc., USA). The sequences of the forward and reverse primers of the 25 TAS2Rs were taken from Liszt *et al.*(13) (Supporting Information Table-SI 3). Verified primers for the TAS1Rs (TAS1R1: qHsaCID0013443; TAS1R2: qHsaCID0016106; TAS1R3: qHsaCED0002321) and for MAPK1 (qHsaCEP0050000) were obtained from Bio-Rad Laboratories. PPIA^37^ and GAPDH^38^ were used as reference genes. The effects of the peptides on gene expression were analyzed in comparison to untreated control cells.

### 2.12 Transient Knock-Down of TAS2R16 or TAS2R38 Expression in HGT-1 Cells

Expression of TAS2R16 and TAS2R38 was reduced by treatment of HGT-1 cells with siRNA. 100,000 cells per well were seeded 1 day before the experiment into a 24-well plate. All reagents and siRNA were purchased from Thermo Fisher Scientific, USA (cytotoxicity was excluded by MTT). Transfection was performed with Lipofectamine RNAiMAX in Opti-Medium according to the manufacturer’s protocol. 1–50 nM of different siRNA sequences (HSS121396 and HSS181763 for TAS2R16 and HSS108754 and HSS108756 for TAS2R38) and three different incubation times (24, 48, and 72 h) were tested. Mock control experiments were performed analogously with 1 or 10 nM Stealth RNAi siRNA negative control. To verify the functionality of the transfection process, verified 10 nM siRNA targeting MAPK1 (VHS40312) was used (positive control). The transfection rate was checked by qPCR as described in section 2.11. For the proton secretion assays, 20,000 cells per well were seeded 1 day before transfection into a black 96-well plate. After 72 h of transfection, proton secretion activities of TAS2R16 knock-down or TAS2R38 knock-down HGT-1 were compared with the mock transfected cells by means of ΔIPX.

### 2.13 Statistical Analysis

All data are presented as mean ± standard error of the mean (SEM) unless otherwise indicated. At least three biological replicates were prepared from each experiment. Statistical analyses of different treatments with the untreated control were performed using the one-way ANOVA Holm-Šidák *post hoc* test or *t*-test Holm-Šidák method, after the Nalimov outlier test. Different *p* values are indicated with asterisks according to the following scheme: * = *p* ≤ 0.05, ** = *p* ≤ 0.01, *** = *p* ≤ 0.001, **** = *p* ≤ 0.0001.

## 3 Results and Discussion

### 3.1 *In Vitro* Digestion under Gastric Conditions Produces Bitter Peptides

Protein digestion in the human stomach is essentially characterized by two important aspects: one is the low pH caused by gastric acid. The other is the presence of the enzyme pepsin, which cleaves peptide bonds.^28^ In order to elucidate the formation of peptides during digestion of casein over a wide time spectrum, samples were taken for identification at seven different time points during a simulated *in vitro* digestion (0–6 h of digestion). All samples were analyzed in four biological replicates in untargeted ToF-MS-IDA mode.

After the first hour, 77.8 ± 9.8 different peptides were identified increasing to 91.3 ± 2.2 after 2 h. At all later time points, no major changes in the number of peptides were detected (3 h: 87.5 ± 9.6, 4 h: 94.0 ± 11.9, 5 h: 93.3 ± 13.5, and 6 h: 97.0 ± 12.9), but a large number of different peptides were found. Comparison of the resulting peptides at all 6 time points resulted in a peptide library of 238 different casein peptides (67 for α_S1_-casein, 21 for α_S2_-casein, 21 for β_A1_-casein, 30 for β_A2_-casein, 62 for β_A1/A2_-casein, and 37 for κ-casein). To exclude possible peptide contamination of the casein used, a sample was also taken before reduction of pH to 3 and addition of the enzyme pepsin (0 h). Here, 2.5 ± 1.1 peptides were identified. To assess if pH change alone caused peptide formation, incubation of casein at pH 3 and 7 without pepsin was carried out over the same time period. This resulted in the formation of 3.6 ± 1.4 (at pH 3) and 4.0 ± 0.8 (at pH 7) peptides within 6 h, respectively. It shows that the low pH alone is not sufficient to release peptides from casein. Nevertheless, the low pH is necessary to ensure the activity of pepsin since it denatures at higher pH values.^28^

To focus on the most important peptide candidates in the next experiments, the following criteria were used for selection. The Andromeda score indicates how closely a spectrum generated in silico matches the measured MS/MS spectra. Above an Andromeda score of 100, the identified peptides match in almost all cases.^39^ Therefore, only peptides with a score above 150 were considered. As a result, peptide selection was limited to the following 11: FVAPFPEVF (α_S1_-CN_24–32_), INNQFLPYPYYAKPAA (κ-CN_51–66_), LTDVENLHLPLPLL (β_A2_-CN_127–140_), PVVVPPFLQPEVM (β_A1/A2_-CN_81–93_), TDVENLHLPLPLL (β_A2_-CN_128–140_), TDVENLHLPLPLLQS (β_A2_-CN_128–142_), VAPFPEVF (α_S1_-CN_25–32_), YFYPEL (α_S1_-CN_144–149_), YQEPVLGPVRGPFPIIV (β_A1/A2_-CN_193–209_), YTDAPSF (α_S1_-CN_173–179_), and YYVPLGTQ (α_S1_-CN_165–172_).

To predict bitterness in the second step, three different prediction tools were applied. First, the *Q* values of the peptides were calculated, indicating the average hydrophobicity.^33,40^ Only one peptide (YTDAPSF; *Q* value = 1323) had a value below 1400 cal/mol and was excluded. The two tools iBitter-SCM^41^ and BERT4Bitter^42^ predict the bitterness of peptides based on their amino acids and their sequence. This allowed the number of peptides to be reduced to six. Although both FVAPFPEVF (iBitter-SCM score 451.3) and VAPFPEVF (iBitter-SCM score 469.3) are predicted to be bitter peptides according to all three tools, FVAPFPEVF was excluded to avoid the study of peptides with overlapping sequences.

The sequences of the five selected peptides are PVVVPPFLQPEVM (β_A1/A2_-CN_81–93_), VAPFPEVF (α_S1_-CN_25–32_), YFYPEL (α_S1_-CN_144–149_), YQEPVLGPVRGPFPIIV (β_A1/A2_-CN_193–209_), and YYVPLGTQ (α_S1_-CN_165–172_). All of these peptides selected were released from α_S1_- or β-casein, with the peptides derived from β-casein found in both natural variants (A1 and A2). The fact that α_S1_- and β-casein represent the majority of casein present in cow’s milk (38.4% and 36.5%) is another aspect in favor of the five selected peptides.^43^

To verify the five selected peptides, synthesized reference peptides were purchased and ultra-high performance liquid-chromatography (UHPLC)–MS/MS-MRM spectra were recorded (Supporting Information Figure-SI 2A). The retention times [ultra-performance liquid chromatography (UPLC)] and SRM mass transitions (MS/MS) of peptides in *in vitro* samples were compared with the previously recorded spectra of externally synthesized peptides. The identification of all five selected peptides was clearly confirmed (Supporting Information Figure-SI 2B). For quality control, reference solutions were analyzed between LC–MS measurements of the samples. The following recovery rates were obtained: PVVVPPFLQPEVM: 102.6 ± 2.2%, VAPFPEVF: 100.0 ± 3.6%, YFYPEL: 97.5 ± 3.7%, YQEPVLGPVRGPFPIIV: 103.5 ± 2.1%, and YYVPLGTQ: 97.2 ± 4.9%.

All five selected peptides have already been described in the literature as cleavage products of casein digestion.^44^ Various bioactivities have already been found for three of the peptides. YQEPVLGPVRGPFPIIV is the best known representative and exhibits antimicrobial^45^ and immunomodulatory abilities^46^ as well as angiotensin-converting-enzyme inhibitory activity.^47^ YFYPEL was found to increase the expression of MUC5AC in human intestinal cells. The resulting increase in the mucus barrier may prevent gastrointestinal diseases.^48^ In addition, transport through Caco-2 cell monolayers was observed for YQEPVLGPVRGPFPIIV and PVVVPPFLQPEVM.^49^

After verifying the formation of the selected peptides in *in vitro* digestion, sensory analyses were performed to confirm their bitter taste. After purity tests by reversed-phase-(HPLC) and quantitative ^1^H NMR, Three AFC tests (*n* = 17–18) of all peptides (1.5 mM in water) were performed against two samples containing water. This showed that all five peptides exhibit a distinct bitter taste (Supporting Information Figure-SI 5, *p* ≤ 0.001). The results of the bitter prediction tools used at the beginning could be confirmed. This demonstrates that bitter peptides were released during the gastric digestion of non-bitter casein.

### 3.2 Monitoring of the Formation of Bitter Peptides PVVVPPFLQPEVM, VAPFPEVF, YFYPEL, YQEPVLGPVRGPFPIIV, and YYVPLGTQ during *In Vitro* Digestion

**Figure 1:**
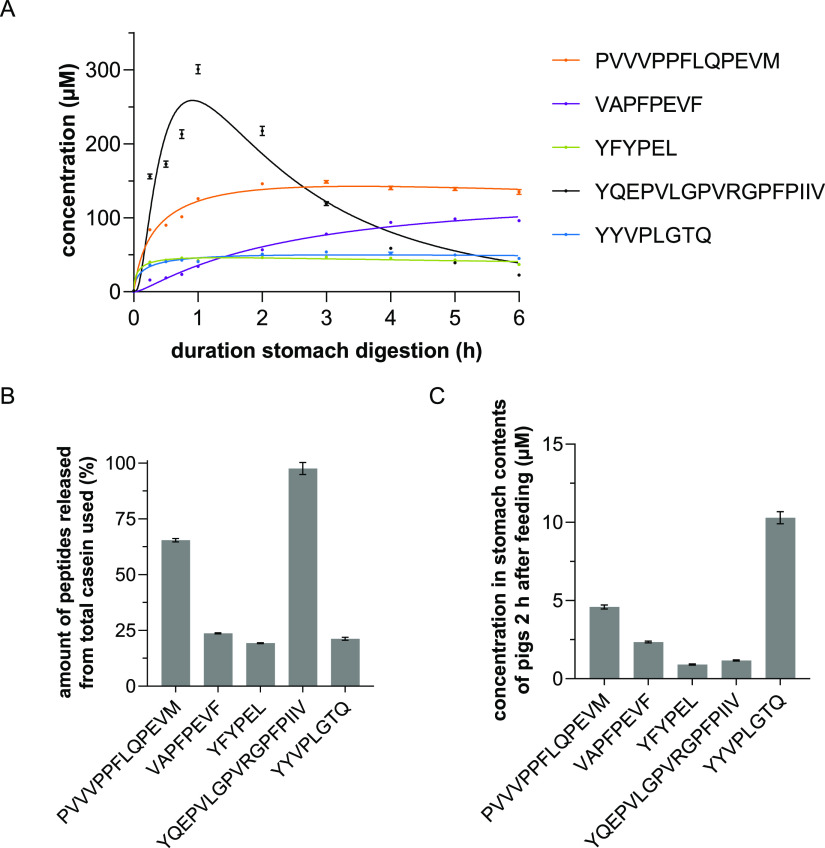
(A) Peptide concentrations in the course of in vitro digestion. Samples were taken after 0, 0.25, 0.5, 0.75, 1, 2, 3, 4, 5, and 6 h of digestion and quantitated by means of targeted UHPLC–MS/MS-MRM measurements. Data shown as mean ± SEM, *n* = 4, transitions per peptide = 5. (B) Release rates of the five investigated peptides related to α_S1_- or β-casein after 2 h of in vitro digestion. Data are shown as mean ± SEM, *n* = 4, transitions per peptide = 5. (C) Concentrations of the five investigated peptides related to α_S1_- or β-casein after 2 h of in vivo digestion. Data are shown as mean ± SEM, *n* = 6, transitions per peptide = 5.

To investigate the formation and degradation of peptides during digestion, their concentrations were determined at different time points. For this purpose, a suitable LC–MS/MS-MRM method was developed. Since the untargeted measurements of the samples showed a high release of different peptides already in the first hour of *in vitro* digestion, a sample was taken every 15 min for quantitation within the first hour (Figure 1A). The *in vitro* digestion was highly reproducible, showing only small deviations between experiments (SEM).

The highest release was observed for the peptides YQEPVLGPVRGPFPIIV (up to 300.8 μM) and PVVVPPFLQPEVM (up to 148.7 μM). While PVVVPPFLQPEVM was not further degraded during the digestion, YQEPVLGPVRGPFPIIV underwent a continuous cleavage over the 6 h, so that its final concentration was lower (22.5 μM) than that of all other peptides analyzed. For example, the peptides YQEPVLG (β_A1/A2_-CN_193–199_) and PVRGPFPIIV (β_A1/A2_-CN_200–209_) were identified as cleavage products (for all peptide fragments found, see Supporting Information Figure-SI 7). The peptides VAPFPEVF, YFYPEL, and YYVPLGTQ were released in lower concentrations at the beginning (between 34.3 and 42.3 μM after 1 h), and there is no noticeable degradation during further digestion, similar to PVVVPPFLQPEVM.

No formation of the five peptides in the control digests at pH 3 and 7 without pepsin could be detected by the targeted measurements.

### 3.3 Selected Peptides Are Also Formed during *In Vivo* Digestion Experiments

To confirm that the peptides formed *in vitro* are also generated *in vivo*, feeding experiments were performed in pigs, as the function of their digestive tract is very similar to humans.^50^ The pH of the stomach contents was 2.9 ± 0.7, close to the *in vitro* conditions. Two hours after administration of casein, the stomach content of the animals was analyzed in six biological replicates by means of LC–MS/MS and LC–ToF-MS in IDA mode as detailed in sections 3.1 and 3.2. This resulted in a peptide library consisting of 270 peptides. All previously identified peptides from the *in vitro* approaches after 2 h (*n* = 4) were found in this library. The high correlation of peptides formed *in vitro* and *in vivo* is consistent with the results described by Egger *et al.*(51)

In particular, the five selected peptides were unambiguously identified in both ToF-MS-IDA and targeted UHPLC–MS/MS-MRM measurements (Supporting Information Figure-SI 2C). The quantitation was also performed analogously to the *in vitro* samples. As the volume of gastric contents varied between 100 and 1000 mL, the concentrations of released peptides were normalized to 100 mL gastric volume. The concentrations of the five peptides ranged from 0.91 ± 0.03 μM for YFYPEL to 10.30 ± 0.38 μM for YYVPLGTQ (Figure 1C). The release rates of the peptides were lower than those during *in vitro* digestion. This could be due to incomplete suspension of the ingested casein.

### 3.4 Determination of Physiological Concentrations of Peptides in the Stomach

In order to study meaningful effects of peptides on human digestion, it is essential to determine concentrations that are actually achievable in the stomach after habitual dietary intake of dairy products. The concentrations of peptides released from α_S1_- and β-casein in *in vitro* and *in vivo* digestion were in a micromolar range. A similar range of casein concentrations can be expected in the human stomach after ingestion of dairy products. This is based on the assumption that 1 L of cow’s milk contains about 27.5 g of casein,^17^ with α_S1_-casein (*m*_w_ = 24.5 kDa; UniProt: P02662) and β-casein (*m*_w_ = 25.1 kDa; UniProt: P02666) accounting for 38.4% and 36.5%, respectively,^43^ resulting in maximum concentrations of 460 μM for α_S1_-casein or 425 μM for β_A1/A2_-casein. However, the actual concentrations are likely to be much lower due to dilution by gastric acid. In addition, pepsin cleaves at different sites within the amino acid sequences, leading to the formation of competing peptides with similar sequences and consequently to a lower release of the peptides under investigation. Depending on the sequence, the peptides were released in variable amounts (related to the respective casein variant). The peptide YQEPVLGPVRGPFPIIV showed the highest release after 2 h with almost 100% (Figure 1B). One reason for this could be the position of the sequence at the C-terminus of β-casein. In addition, pepsin preferably cleaves between tyrosine and leucine.^29^ The release rates of the other peptides ranged from 19.3% (YFYPEL) to 65.5% (PVVVPPFLQPEVM). In the case of YQEPVLGPVRGPFPIIV, further degradation took place during the course of digestion, resulting in low concentrations of parental peptides. For these reasons, peptide concentrations between 0.01 and 200 μM were chosen for further experiments.

### 3.5 Effect of Casein-Derived Bitter Peptides on Mechanisms Regulating Gastric Acid Secretion by HGT-1 Cells

To cover the range of physiological concentrations of the selected peptides possible in the human stomach, peptide concentrations between 0.01 and 200 μM were chosen as described above. Cell viability after incubation with the peptides, hydrolysate, and intact casein was tested before (≥97.5% compared to control).

**Figure 2:**
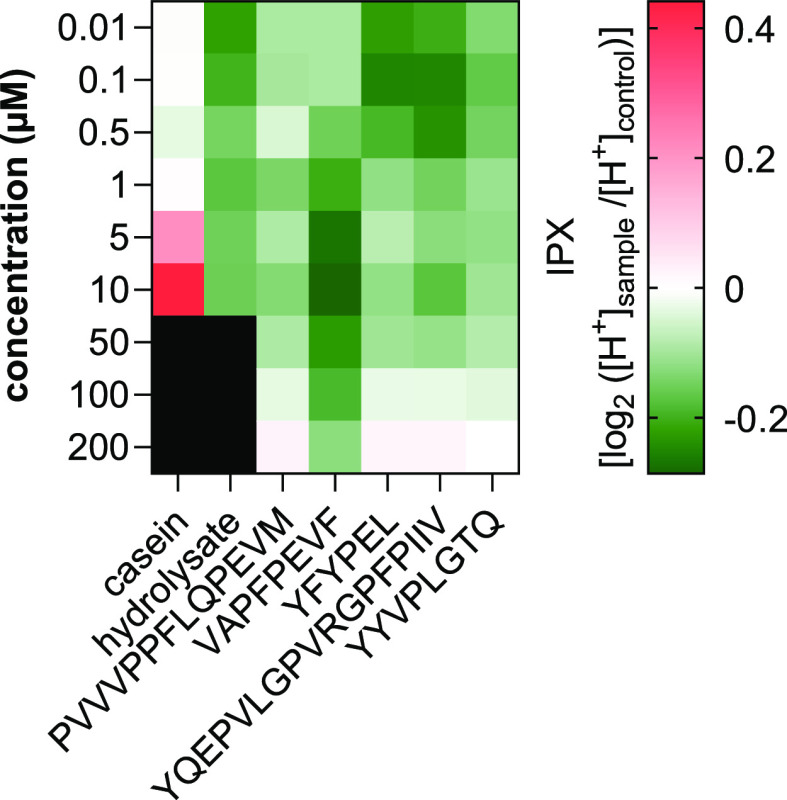
Heatmap showing the change in mean secretory activity (IPX) in HGT-1 cells on incubation with casein (equimolar related to the relevant forms α_S1_- and β-casein), casein-hydrolysate (equimolar related to the relevant forms α_S1_- and β-casein; after 1 h of gastric digestion), and the five selected peptides PVVVPPFLQPEVM, VAPFPEVF, YFYPEL, YQEPVLGPVRGPFPIIV, and YYVPLGTQ at concentrations between 0.01 and 10 or 200 μM. Red shades represent inhibition of activity (positive IPX values) and green shades represent stimulation of proton secretion (negative IPX values). Data are shown as mean after incubation for 10 min, *n* = 4–8, t.r. = 4–6.

In order to check if intact casein (before digestion) already has an effect on the proton secretion of HGT-1 cells, the impact of 0.01–10 μM casein (equimolar related to the relevant forms α_S1_- and β-casein) was analyzed by proton secretion assay. The intercellular proton index IPX indicates the secretory activity. Negative values represent increased secretion of protons and therefore stimulation of mechanisms regulating gastric acid secretion in HGT-1 cells. In contrast, positive values represent an inhibition of secretions compared to the untreated control. No significant change in mean IPX was found for casein concentrations below 5 μM (Figure 2). Treatment of the HGT-1 cells with casein concentrations of 5 μM (ΔIPX = +0.212 ± 0.029; *p* ≤ 0.001) and 10 μM (ΔIPX = +0.441 ± 0.037; *p* ≤ 0.0001) inhibited proton secretion. This shows that intact casein at low concentrations has no effect on mechanisms regulating gastric acid secretion by HGT-1 cells, whereas higher concentrations have a regulatory effect and inhibit secretion. Investigation of the effects of higher concentrations was not possible due to the poor solubility of casein.

To test the effects of peptides produced during digestion on proton secretion, cells were incubated with 0.01–10 μM casein hydrolysate (after 1 h of gastric digestion). It was found that concentrations between 5 and 10 μM did not inhibit mechanisms regulating gastric acid secretion (Figure 2). At concentrations of 0.01 and 0.1 μM hydrolysate, significant (*p* ≤ 0.05) stimulation of secretion was detected, with ΔIPX changes of −0.222 ± 0.046 and −0.199 ± 0.055, respectively. Consequently, the peptides produced during gastric digestion of non-bitter casein as a mixture have a stimulating effect on mechanisms regulating gastric acid secretion, suggesting that bitter-tasting peptides, among others, were released. For all five selected peptides, a significant hormetic concentration-dependent influence on the secretory activity was found. Holik *et al.*(52) showed a hormetic dose–response when HGT-1 cells were incubated with l-arginine. HGT-1 cells released more serotonin when treated with lower l-arginine concentrations (10 mM) than with higher l-arginine concentrations (50 mM). This effect was additionally found upon serotonin-induced stimulation of proton secretion from HGT-1 cells.

While significant ΔIPX was analyzed for the peptide PVVVPPFLQPEVM at 1 and 10 μM only, the other four peptides showed a significant increase in secretory activities of HGT-1 cells over a wider concentration range. Incubation with VAPFPEVF at a concentration of 10 μM showed the highest change in ΔIPX with −0.286 ± 0.037 (*p* ≤ 0.0001; Supporting Information Figure-SI 3). In addition, also the peptides YFYPEL (0.1 μM; ΔIPX −0.253 ± 0.027; *p* ≤ 0.0001), YQEPVLGPVRGPFPIIV (0.1 μM; ΔIPX −0.203 ± 0.048; *p* ≤ 0.0001), and YYVPLGTQ (0.1 μM; ΔIPX −0.166 ± 0.017; *p* ≤ 0.0001) stimulated the secretion of protons (Figure 2).

Interestingly, the IPX profiles of YFYPEL and YQEPVLGPVRGPFPIIV were very similar. Despite different lengths, different side chains, and different origins (YFYPEL (α_S1_-CN_144–149_) and YQEPVLGPVRGPFPIIV (β_A1/A2_-CN_193–209_)), both peptides show great similarity in concentration-dependent stimulating mechanisms, regulating gastric acid secretion in HGT-1 cells (Supporting Information Figure-SI 3).

Overall, mechanisms regulating proton secretion by HGT-1 cells were not affected by low concentrations of casein (<5 μM), whereas they were inhibited at concentrations of 5 μM and higher compared to untreated cells. In contrast, the peptide mixture consisting of casein hydrolyzed by pepsin already demonstrated stimulatory effects on the mechanisms regulating proton secretion by HGT-1 cells. This was consistent with the result that bitter peptides were also produced during the digestion process, which cause even greater stimulation of proton secretion when administered in their isolated forms. The three peptides with the greatest effects on proton secretory activity (VAPFPEVF, YFYPEL, and YQEPVLGPVRGPFPIIV) were selected to investigate their effects on taste (TAS1R) and bitter (TAS2R) receptor gene expression.

### 3.6 Bitter Peptides Affect the Expression of Various Bitter Receptors, Especially TAS2R16 and TAS2R38

**Figure 3:**
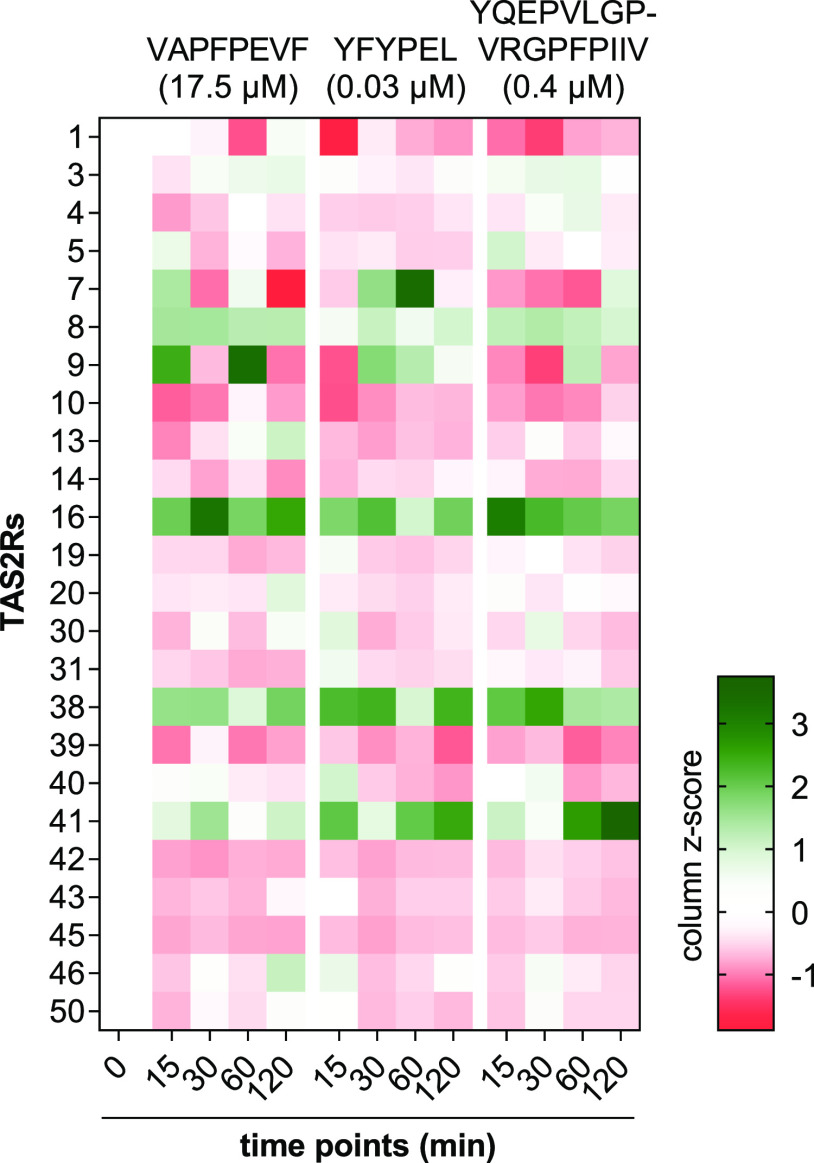
Auto-scaled changes (column z-score) in gene expressions of 24 bitter receptors at each time point found in HGT-1 cells after incubation for 15/30/60/120 min with VAPFPEVF (17.5 μM), YFYPEL (0.03 μM), or YQEPVLGPVRGPFPIIV (0.4 μM). Normalized to the expression of PPIA and GAPDH (reference genes). Data are shown as mean, *n* = 3–5, t.r. = 3.

**Figure 4:**
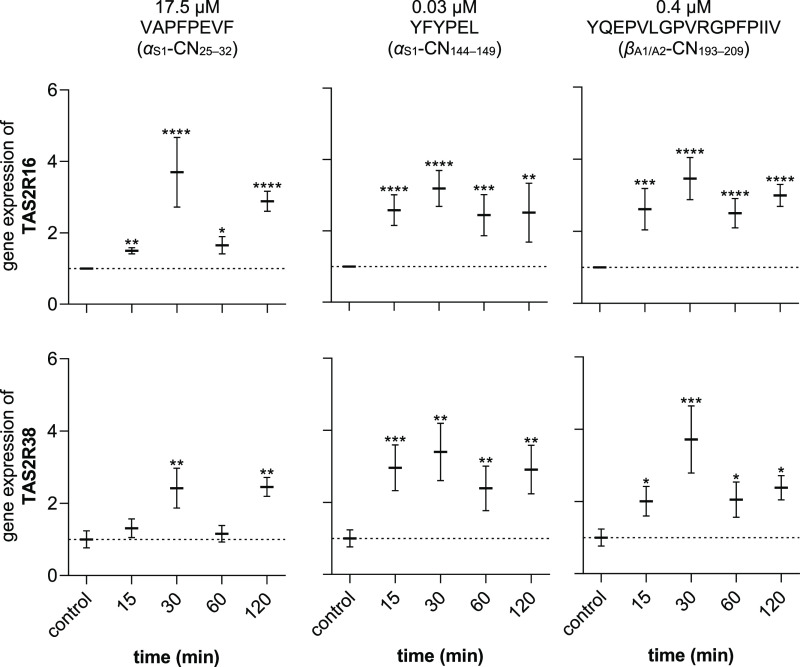
Changes in gene expressions (fold change) of bitter receptors TAS2R16 (top) and TAS2R38 (bottom) after incubation with peptides VAPFPEVF (17.5 μM; left), YFYPEL (0.03 μM; center), and YQEPVLGPVRGPFPIIV (0.4 μM; right) for 15/30/60/120 min. Normalized to the expression of PPIA and GAPDH (reference genes). Data are shown as mean ± SEM, *n* = 3–5, t.r. = 3, statistics: *t*-test Holm-Šidák method; significant differences are expressed with * = *p* ≤ 0.05, ** = *p* ≤ 0.01, *** = *p* ≤ 0.001, **** = *p* ≤ 0.0001.

To determine which peptide concentrations between the tested 0.01–200 μM have the highest impact on secretory activity, a curve fit calculation was performed for each peptide (Supporting Information Figure-SI 4). The minima obtained represent the respective concentrations with the lowest IPX values, thereby showing highest impact on secretory activity. Incubation of HGT-1 cells with VAPFPEVF (17.5 μM), YFYPEL (0.03 μM), and YQEPVLGPVRGPFPIIV (0.4 μM) resulted in both up- and down-regulation of bitter receptor gene expressions at all four time points investigated (Figure 3 and Supporting Information Table-SI 1). Expression of *TAS2R60* could not be detected in HGT-1 cells, either with or without treatment. The change in gene expression for *TAS2R16* and *TAS2R38* was differently affected (Figure 4). *TAS2R41* showed variable upregulation after treatment with the peptides but did not reach statistical significance (Figure 3).

For *TAS2R16*, an upregulation was found at all time points and for all three peptides (*p* ≤ 0.05). The highest fold changes in the regulation of *TAS2R16* were found for VAPFPEVF (3.70 ± 0.97, *p* < 0.0001, df = 16, *t* = 5.4), YFYPEL (3.19 ± 0.50, *p* < 0.0001, df = 16, *t* = 6.3), and YQEPVLGPVRGPFPIIV (3.46 ± 0.58, *p* < 0.0001, df = 16, *t* = 7.4), respectively, after an incubation with the respective peptide of 30 min (Figure 4, top). Since all three peptides caused an upregulation (*p* ≤ 0.05) of gene expression of the *TAS2R16* at all time points between 15 min and 2 h, it was hypothesized that this receptor plays a crucial role in the increased secretion of gastric acid in HGT-1 cells incubated with bitter peptides. It has already been observed that *TAS2R16*, among other bitter receptors, is targeted by peptides from casein hydrolysates.^19^

In addition, upregulation of *TAS2R38* was also observed. For peptides with similar IPX profiles YFYPEL and YQEPVLGPVRGPFPIIV, this regulation was also significant (*p* ≤ 0.05) at all time points (Figure 4, bottom). When HGT-1 cells were incubated with the peptide VAPFPEVF, no significant changes in gene regulation of *TAS2R38* could be detected after 15 and 60 min, while this was the case after 30 (2.42 ± 0.55, *p* < 0.01, df = 15, *t* = 3.1) and 120 min (2.45 ± 0.26, *p* < 0.01, df = 15, *t* = 3.8).

The most effective upregulation of gene expression for each peptide was observed for VAPFPEVF on *TAS2R16*, as described above, for YFYPEL (after 60 min, fold change 6.71 ± 1.56, *p* < 0.05, df = 14, *t* = 2.6) in TAS2R7 and for YQEPVLGPVRGPFPIIV (after 120 min, fold change 4.61 ± 2.03, *p* ≤ 0.01, df = 14, *t* = 3.1) in *TAS2R41*. The most pronounced down-regulation was found for *TAS2R10* for all three peptides after incubation with VAPFPEVF after 15 min (0.34 ± 0.05, *p* < 0.0001, df = 16, *t* = 6.1), with YFYPEL after 30 min (0.41 ± 0.05, *p* < 0.001, df = 16, *t* = 4.2), and with YQEPVLGPVRGPFPIIV after 15 min (0.52 ± 0.06, *p* < 0.01, df = 16, *t* = 4.0) (Supporting Information Table-SI 1).

Taken together, it was found that the gene expression of *TAS2R16* and *TAS2R38* was significantly upregulated after only 15 min of incubation in the case of almost all three bitter peptides. This upregulation became even stronger after 30 min. This indicates that these two receptors play a decisive role in the mechanism of gastric acid secretion from HGT-1 cells, when they are treated with bitter peptides. For TAS2R16, a high specificity for glycosides has been found in the past,^53^ whereas TAS2R38 is known for the perception of phenylthiocarbamides.^54,55^ However, no prior knowledge existed regarding the effect of the peptides identified here on TAS2R16 and TAS2R38. To validate the involvement of these two receptors, knock-down experiments were performed in HGT-1 cells.

### 3.7 Peptides Slightly Affect the Expression of Taste Receptors TAS1R

Proton secretion has been shown in the past to be affected not only by bitter substances involving TAS2Rs but also by sweeteners.^56^ For this reason, the gene expression of TAS1R1, TAS1R2, and TAS1R3 was investigated. While the receptors responsible for umami taste, TAS1R1 and TAS1R3, could be detected in HGT-1 cells, no gene expression of TAS1R2 was found. Consequently, no heterodimer of TAS1R2 and TAS1R3 can be formed and, therefore, no sweet taste sensing can be hypothesized by HGT-1 cells, as previously reported.^56^ After incubation of HGT-1 cells with the selected peptides, a significant increase (*p* < 0.0001, df = 16, *t* = 5.6) in the expression of TAS1R3 was found only for 0.03 μM YFYPEL after 15 min and a decrease (*p* < 0.001, df = 16, *t* = 4.3) in the expression of TAS1R1 after 30 min (Supporting Information Figure-SI 6, center). No significant changes in TAS1R1 and TAS1R3 expression were detected after 60 and 120 min. Also, peptides VAPFPEVF (17.5 μM) and YQEPVLGPVRGPFPIIV (0.4 μM) had no significant effect on the expression (Supporting Information Figure-SI 6). Overall, the changes in the expression of *TAS1R1* and *TAS1R3* were minor compared to those of the bitter receptor genes and, presumably, of only minor biological significance.

### 3.8 Impact of TAS2R16 and TAS2R38 on Peptide-Induced Stimulation of Proton Secretion in HGT-1 Cells

**Figure 5:**
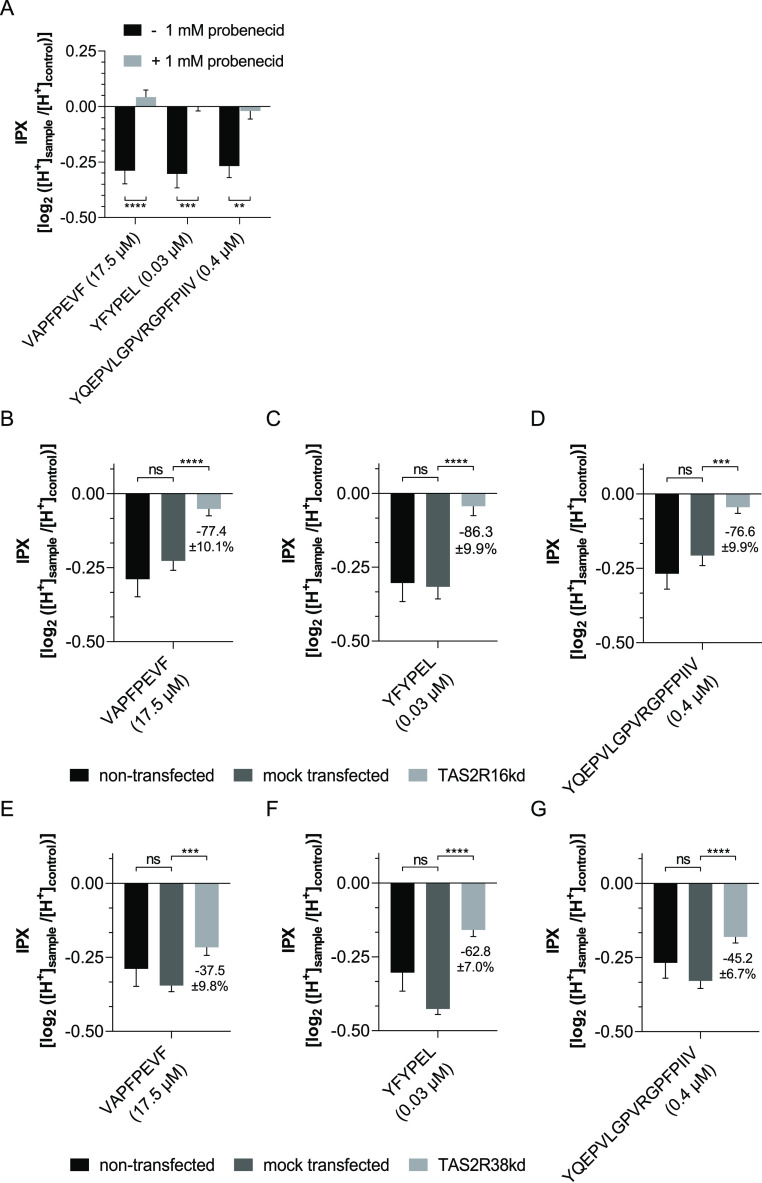
Effect on proton secretion after incubation with VAPFPEVF (17.5 μM), YFYPEL (0.03 μM), and YQEPVLGPVRGPFPIIV (0.4 μM), respectively, of (A) HGT-1 cells with (light gray bars) and without (black bars) 1 mM probenecid; (B–D) non-transfected (black bars), mock-transfected (dark gray bars), and TAS2R16kd (light gray bars) HGT-1 cells; and (E–G) non-transfected (black bars), mock-transfected (dark gray bars), and TAS2R38kd (light gray bars) HGT-1 cells. Data are shown as mean ± SEM after incubation for 10 min, *n* = 4–8, t.r. = 4–12, control: KRHB, statistics: one-way ANOVA Holm-Šidák post hoc test; significant differences are expressed with ** = *p* ≤ 0.01, *** = *p* ≤ 0.001, **** = *p* ≤ 0.0001.

To verify the involvement of TAS2R16 and TAS2R38 in the increased proton secretion after incubation of HGT-1 cells with the bitter peptides, the cells were co-treated with 1 mM of the TAS2R16 and TAS2R38 antagonist probenecid (cell viability ≥ 97.5% compared to control). Probenecid is known to selectively inhibit TAS2R16, TAS2R38, and TAS2R43.^57^ HGT-1 cells treated with the bitter peptides alone (VAPFPEVF (17.5 μM), YFYPEL (0.03 μM), and YQEPVLGPVRGPFPIIV (0.4 μM)) showed increased proton secretion compared to untreated control cells (*p* < 0.01). Co-incubation with probenecid (1 mM) reduced this stimulation back to baseline levels (for VAPFPEVF ΔIPX = +0.043 ± 0.031; *p* > 0.92; for YFYPEL ΔIPX = −0.001 ± 0.019; *p* > 0.99; for YQEPVLGPVRGPFPIIV ΔIPX = −0.020 ± 0.036; *p* > 0.99), so that no changes in proton secretion could be detected anymore compared to untreated cells (Figure 5A).

To confirm the respective involvement of TAS2R16 and TAS2R38 in the increased proton secretion by HGT-1 cells, knock-down experiments were performed. The highest knock-down efficiency was observed by RT-qPCR after 72 h of transfection with 10 nM siRNA targeting *TAS2R16* (HSS121396) and 1 nM siRNA targeting *TAS2R38* (HSS108754), respectively. The expression of *TAS2R16* was reduced to 42.2 ± 1.7% (*p* < 0.001) and to 62.8 ± 10.7% (*p* < 0.01) for *TAS2R38* (Supporting Information Figure-SI 6). Cytotoxic effects of siRNA were excluded before performing the experiments (≥97.5% compared to control). Cell viability after transfection was 93.2 ± 1.2% (compared to DMEM control), which is consistent with the manufacturer’s data. To monitor the transfection process, the expression of MAPK1 was reduced to 60.2 ± 3.9% (*p* < 0.01).

To investigate the involvement of TAS2R16 in the increased proton secretion activity induced by incubation with the peptides, the proton secretion assays were repeated with TAS2R16kd and mock transfected HGT-1 cells. This showed that the increase in proton secretion activity in TAS2R16kd cells was decreased by 77.4 ± 10.1% for VAPFPEVF (17.5 μM, *p* < 0.0001), by 86.3 ± 9.9% for YFYPEL (0.03 μM, *p* < 0.0001), and by 76.6 ± 9.9% for YQEPVLGPVRGPFPIIV (0.4 μM, *p* < 0.001) (Figure 5B). Analogously, the stimulatory effect of bitter peptides in TAS2R38kd HGT-1 cells also decreased. Stimulation by incubation with VAPFPEVF was reduced by 37.5 ± 9.8% (17.5 μM, *p* < 0.001), with YFYPEL by 62.8 ± 7.0% (0.03 μM, *p* < 0.0001), and with YQEPVLGPVRGPFPIIV by 45.2 ± 6.7% (0.4 μM, *p* < 0.0001) (Figure 5C).

Mock-transfected cells showed no significant differences from non-transfected cells in both cases. TAS2R-independent histamine-induced stimulation of proton secretion does not differ from non-transfected cells in either mock-transfected or TAS2R16 or TAS2R38 knockdown HGT-1 cells (Supporting Information Figure-SI 9). This shows that the secretory activity of the cells was not affected by transfection, except for the receptor in question.

The above results indicate that both TAS2R16 and TAS2R38 play a functional role in the increased proton secretion by HGT-1 cells exposed to the bitter peptides tested. Therefore, we conclude that bitter peptides released from casein during gastric digestion modulate digestive processes, namely, proton secretion activity, involving TAS2R16 and TAS2R38. Since proton secretion stimulated by bitter peptides was reduced to a greater extent in TAS2R16kd than in TAS2R38kd HGT-1 cells, it can be assumed that although both receptors are involved in the mechanism, TAS2R16 may be of higher functional importance. However, one has to note that differences in the effect size may also be caused by the different transfection efficiencies, as the gene expression of TAS2R16 was reduced by 20% more than that of TAS2R38.

A response of TAS2R16 to peptides in casein hydrolysates has already been detected by Maehashi *et al.*(19) Here, HEK293 cells expressing either TAS2R1, TAS2R4, TAS2R14, or TAS2R16 responded to casein hydrolysates. While activation of TAS2R1, TAS2R4, and TAS2R14 by amino acids and peptides could be confirmed by Kohl *et al.*, no unambiguous peptide sequences leading to activation of either TAS2R16 or TAS2R38 have yet been identified.^20^ Our results indicate that both TAS2R16 and TAS2R38 play functional roles in the increased proton secretion in HGT-1 cells by the tested bitter peptides with clearly identified sequences at physiologically achievable concentrations.

In conclusion, our results demonstrate that bitter peptides are released from the non-bitter protein casein during gastric digestion. While intact casein had no or higher concentrations of inhibitory effect on proton secretion, representing a key mechanism of gastric acid secretion of HGT-1 cells, casein hydrolysate induced a stimulation. This effect was further enhanced upon treatment with isolated bitter peptides. While qPCR data suggest involvement of TAS2R16 and TAS2R38, co-incubation experiments with the antagonist probenecid showed that by blocking both receptors, no significant stimulation of mechanisms regulating gastric acid secretion was measurable, indicating a functional role of TAS2R16 and TAS2R38. These results were confirmed by knock-down experiments in which the gene expression of *TAS2R16* and *TAS2R38* was reduced by means of siRNA. Therefore, we verified a functional role of TAS2R16 and TAS2R38 in the bitter peptide-mediated stimulation of proton secretion in HGT-1 cells. This implicates a role of bitter peptides released during gastric cleavage from non-bitter-tasting proteins on gastric response mechanisms regulating digestion and food intake. Future clinical trials are warranted to determine respective effect sizes in human subjects in order to fully elucidate the potential of such peptides to modulate food intake and help to maintain a healthy body weight.

## Supporting Information Available

Changes in gene expression of the bitter receptors after incubation with peptides, optimized MS parameters for each peptide and transition, sequences of the primer pairs used for RT-qPCR, illustration of the proposed mechanism of proton secretion induced by bitter compounds in HGT-1 cells, comparison of retention times (UPLC) and SRM mass transitions (MS/MS) of the five peptides, effect on the proton secretion of HGT-1 cells incubated with VAPFPEVF, YFYPEL, and YQEPVLGPVRGPFPIIV, curve fit calculations of the proton secretion profile, results of sensory experiments, changes in gene expression of the TAS1Rs after incubation with peptides, identified degradation products of YQEPVLGPVRGPFPIIV, results of transient transfection, and results of histamine-induced stimulation of proton secretion of non-transfected and transfected HGT-1 cells (PDF)
