# Supplementary material for: Bitter Peptides YFYPEL, VAPFPEVF, and YQEPVLGPVRGPFPIIV, Released during Gastric Digestion of Casein, Stimulate Mechanisms of Gastric Acid Secretion via Bitter Taste Receptors TAS2R16 and TAS2R38
Source: J Agric Food Chem. 2022 Sep 2;70(37):11591–602. doi: 10.1021/acs.jafc.2c05228 (PMC9501810; doi:10.1021/acs.jafc.2c05228)
Supplement: Supplementary file 1 — jf2c05228_si_001.pdf [file jf2c05228_si_001.pdf]

Supporting Information

Bitter peptides YFYPEL, VAPFPEVF and YQEPVLGPVRGPFPIIV, released during gastric digestion of casein, stimulate mechanisms of gastric acid secretion via bitter taste receptors TAS2R16 and TAS2R38

Phil Richter[1], Karin Sebald[1], Konrad Fischer[2], Maik Behrens[1], Angelika Schnieke[2], Veronika Somoza[1,3,4*]

Tables

Table-SI 1. Changes in gene expression of the bitter receptors after incubation for 15/30/60/120 min with peptides VAPFPEVF (17.5 µM), YFYPEL (0.03 µM) and YQEPVLGPVRGPFPIIV (0.4 µM). Normalized to the expression of PPIA and GAPDH and expression of the receptors in HGT-1 cells without treatment. Data shown as mean ± SEM, n = 3 – 5, t. r. = 3, Statistics: t test Holm-Šidák method; significant differences are expressed with * = p ≤ 0.05.

receptor	treatment	15 min	30 min	60 min	120 min
TAS2R1	VAPFPEVF	0.79 ± 0.12	1.36 ± 0.20	0.44 ± 0.22	1.62 ± 0.61
	YFYPEL	0.36 ± 0.25	1.03 ± 0.38	0.55 ± 0.33	0.73 ± 0.29
	YQEPVLGPVRGPFPIIV	0.33 ± 0.43	0.43 ± 0.23	1.00 ± 0.63	0.84 ± 0.26
TAS2R3	VAPFPEVF	**0.70 ± 0.05***	**1.56 ± 0.22***	1.06 ± 0.04	**1.72 ± 0.12***
	YFYPEL	**1.54 ± 0.07***	1.08 ± 0.16	1.19 ± 0.12	**1.34 ± 0.08***
	YQEPVLGPVRGPFPIIV	1.03 ± 0.11	**1.89 ± 0.19***	**1.61 ± 0.22***	**1.40 ± 0.02***
TAS2R4	VAPFPEVF	**0.50 ± 0.06***	1.14 ± 0.19	0.95 ± 0.07	**1.42 ± 0.14***
	YFYPEL	**1.24 ± 0.07***	0.81 ± 0.12	0.92 ± 0.07	1.13 ± 0.11
	YQEPVLGPVRGPFPIIV	0.79 ± 0.08	**1.71 ± 0.19***	**1.60 ± 0.22***	**1.21 ± 0.03***
TAS2R5	VAPFPEVF	0.91 ± 0.04	1.05 ± 0.07	0.93 ± 0.06	1.23 ± 0.10
	YFYPEL	**1.34 ± 0.03***	1.03 ± 0.10	0.90 ± 0.04	1.02 ± 0.04
	YQEPVLGPVRGPFPIIV	1.25 ± 0.10	**1.45 ± 0.10***	**1.39 ± 0.13***	**1.22 ± 0.03***
TAS2R7	VAPFPEVF	1.20 ± 0.32	0.71 ± 0.34	**1.05 ± 1.05**	0.62 ± 0.21
	YFYPEL	1.23 ± 0.83	2.50 ± 1.32	**6.71 ± 1.56***	1.18 ± 0.41
	YQEPVLGPVRGPFPIIV	0.49 ± 0.24	0.74 ± 0.35	0.70 ± 0.94	1.82 ± 1.25
TAS2R8	VAPFPEVF	1.24 ± 0.13	**2.25 ± 0.32***	1.34 ± 0.16	**2.04 ± 0.23***
	YFYPEL	**1.62 ± 0.22***	**1.90 ± 0.31***	**1.87 ± 0.39***	**1.69 ± 0.21***
	YQEPVLGPVRGPFPIIV	1.39 ± 0.23	**2.45 ± 0.28***	**1.87 ± 0.32***	**1.93 ± 0.23***
TAS2R9	VAPFPEVF	1.73 ± 0.08	1.08 ± 0.47	2.31 ± 0.75	0.99 ± 0.69
	YFYPEL	0.66 ± 0.42	2.64 ± 0.57	3.01 ± 0.75	1.41 ± 0.22
	YQEPVLGPVRGPFPIIV	0.43 ± 0.68	0.44 ± 0.80	1.91 ± 1.12	0.73 ± 0.32
TAS2R10	VAPFPEVF	**0.34 ± 0.05***	0.76 ± 0.14	0.91 ± 0.15	1.15 ± 0.10
	YFYPEL	**0.64 ± 0.08***	**0.41 ± 0.05***	0.72 ± 0.20	0.90 ± 0.11
	YQEPVLGPVRGPFPIIV	**0.52 ± 0.06***	0.78 ± 0.07	0.90 ± 0.12	1.04 ± 0.08
TAS2R13	VAPFPEVF	**0.44 ± 0.09***	1.28 ± 0.33	1.01 ± 0.05	**1.92 ± 0.36***
	YFYPEL	1.14 ± 0.07	**0.53 ± 0.11***	0.78 ± 0.06	0.88 ± 0.36
	YQEPVLGPVRGPFPIIV	0.70 ± 0.11	**1.65 ± 0.32***	1.16 ± 0.15	1.31 ± 0.08
TAS2R14	VAPFPEVF	**0.67 ± 0.02***	0.97 ± 0.05	**0.86 ± 0.03***	1.08 ± 0.03
	YFYPEL	**1.11 ± 0.01***	0.92 ± 0.05	0.99 ± 0.02	**1.21 ± 0.09***
	YQEPVLGPVRGPFPIIV	**0.85 ± 0.04***	1.08 ± 0.06	1.03 ± 0.04	**1.08 ± 0.02***
TAS2R16	VAPFPEVF	**1.50 ± 0.08***	**3.70 ± 0.97***	**1.65 ± 0.24***	**2.88 ± 0.28***
	YFYPEL	**2.58 ± 0.43***	**3.19 ± 0.50***	**2.44 ± 0.58***	**2.51 ± 0.83***
	YQEPVLGPVRGPFPIIV	**2.61 ± 0.58***	**3.46 ± 0.58***	**2.50 ± 0.41***	**3.00 ± 0.31***
TAS2R19	VAPFPEVF	**0.66 ± 0.07***	1.22 ± 0.17	**0.69 ± 0.02***	**1.25 ± 0.09***
	YFYPEL	**1.60 ± 0.02***	0.82 ± 0.09	0.79 ± 0.07	1.05 ± 0.14
	YQEPVLGPVRGPFPIIV	0.85 ± 0.14	**1.58 ± 0.09***	1.27 ± 0.17	1.05 ± 0.03
TAS2R20	VAPFPEVF	**0.70 ± 0.07***	**1.33 ± 0.13***	**0.87 ± 0.03***	**1.78 ± 0.20***
	YFYPEL	**1.37 ± 0.05***	0.93 ± 0.07	0.94 ± 0.09	1.16 ± 0.13
	YQEPVLGPVRGPFPIIV	0.95 ± 0.08	**1.42 ± 0.06***	**1.38 ± 0.08***	**1.31 ± 0.06***
TAS2R30	VAPFPEVF	**0.56 ± 0.09***	**1.53 ± 0.32***	**0.75 ± 0.04***	**1.62 ± 0.19***
	YFYPEL	**1.79 ± 0.06***	**0.62 ± 0.09***	0.89 ± 0.08	1.15 ± 0.31
	YQEPVLGPVRGPFPIIV	0.74 ± 0.13	**1.88 ± 0.20***	1.20 ± 0.17	0.89 ± 0.03
TAS2R31	VAPFPEVF	**0.66 ± 0.05***	1.14 ± 0.14	**0.70 ± 0.04***	**1.22 ± 0.07***
	YFYPEL	**1.65 ± 0.05***	0.91 ± 0.06	0.96 ± 0.05	1.09 ± 0.11
	YQEPVLGPVRGPFPIIV	0.86 ± 0.12	**1.43 ± 0.11***	**1.33 ± 0.12***	0.99 ± 0.04

receptor	treatment	15 min	30 min	60 min	120 min
TAS2R38	VAPFPEVF	1.31 ± 0.26	**2.42 ± 0.55***	1.15 ± 0.23	**2.45 ± 0.26***
	YFYPEL	**2.96 ± 0.64***	**3.40 ± 0.80***	**2.39 ± 0.62***	**2.91 ± 0.67***
	YQEPVLGPVRGPFPIIV	**2.01 ± 0.41***	**3.71 ± 0.93***	**2.05 ± 0.49***	**2.38 ± 0.34***
TAS2R39	VAPFPEVF	**0.39 ± 0.13***	1.37 ± 0.34	**0.55 ± 0.06***	1.16 ± 0.25
	YFYPEL	1.21 ± 0.16	**0.42 ± 0.09***	**0.62 ± 0.12***	0.45 ± 0.31
	YQEPVLGPVRGPFPIIV	**0.53 ± 0.12***	1.16 ± 0.14	0.73 ± 0.13	**0.53 ± 0.09***
TAS2R40	VAPFPEVF	0.81 ± 0.15	1.54 ± 0.37	0.88 ± 0.06	1.42 ± 0.28
	YFYPEL	**1.90 ± 0.12***	0.82 ± 0.12	**0.59 ± 0.06***	0.75 ± 0.14
	YQEPVLGPVRGPFPIIV	0.92 ± 0.14	**1.78 ± 0.29***	0.96 ± 0.26	0.86 ± 0.07
TAS2R41	VAPFPEVF	0.95 ± 0.27	2.32 ± 1.06	0.98 ± 0.20	1.90 ± 0.68
	YFYPEL	**2.82 ± 0.64***	1.56 ± 0.32	**4.58 ± 2.91***	3.00 ± 2.14
	YQEPVLGPVRGPFPIIV	1.31 ± 0.67	1.71 ± 2.35	2.95 ± 1.80	**4.61 ± 2.03***
TAS2R42	VAPFPEVF	**0.52 ± 0.09***	0.88 ± 0.10	**0.71 ± 0.03***	1.20 ± 0.14
	YFYPEL	1.17 ± 0.09	**0.55 ± 0.12***	**0.69 ± 0.11***	0.93 ± 0.09
	YQEPVLGPVRGPFPIIV	0.62 ± 0.19	**1.38 ± 0.15***	1.18 ± 0.13	0.94 ± 0.06
TAS2R43	VAPFPEVF	**0.57 ± 0.07***	1.14 ± 0.25	**0.72 ± 0.03***	**1.49 ± 0.15***
	YFYPEL	**1.49 ± 0.04***	**0.64 ± 0.06***	0.91 ± 0.04	1.02 ± 0.21
	YQEPVLGPVRGPFPIIV	**0.69 ± 0.09***	**1.44 ± 0.12***	1.15 ± 0.14	0.88 ± 0.04
TAS2R45	VAPFPEVF	**0.52 ± 0.05***	1.08 ± 0.17	**0.68 ± 0.03***	1.17 ± 0.11
	YFYPEL	**1.15 ± 0.05***	**0.54 ± 0.06***	**0.70 ± 0.03***	0.94 ± 0.21
	YQEPVLGPVRGPFPIIV	**0.63 ± 0.09***	**1.25 ± 0.12***	1.06 ± 0.12	**0.84 ± 0.04***
TAS2R46	VAPFPEVF	**0.62 ± 0.07***	**1.47 ± 0.29***	0.85 ± 0.05	**1.95 ± 0.21***
	YFYPEL	**1.70 ± 0.08***	0.73 ± 0.12	1.01 ± 0.11	1.30 ± 0.30
	YQEPVLGPVRGPFPIIV	0.69 ± 0.13	**1.73 ± 0.18***	1.30 ± 0.17	1.06 ± 0.04
TAS2R50	VAPFPEVF	**0.56 ± 0.06***	1.39 ± 0.35	0.83 ± 0.05	**1.56 ± 0.18***
	YFYPEL	**1.50 ± 0.06***	0.70 ± 0.11	0.92 ± 0.09	0.91 ± 0.25
	YQEPVLGPVRGPFPIIV	**0.67 ± 0.08***	**1.66 ± 0.19***	1.21 ± 0.13	1.07 ± 0.08
TAS2R60	VAPFPEVF		not detected		
	YFYPEL		not detected		
	YQEPVLGPVRGPFPIIV		not detected		

Table-SI 2. Optimized parameters declustering potential (DP), collision energy (CE), and collision cell exit potential (CXP) for each peptide and transition.

peptide sequence	Q1 mass (Da)	DP (volts)	Q3 mass (Da)	CE (volts)	CXP (volts)
			700.2	9	28
			571.2	13	24
YFYPEL	416.356	36	474.1	13	28
			358.1	11	22
			350.7	11	12
			1058.5	15	26
			978.5	23	28
PVVVPPFLQPEVM	726.897	180	977.5	21	26
			652.2	17	20
			475.2	21	30
			794.3	11	32
			636.4	11	18
YYVPLGTQ	470.592	21	614.2	15	20
			596.3	19	38
			515.2	13	16
			740.4	11	20
			735.3	11	16
VAPFPEVF	453.135	20	641.2	13	14
			570.2	15	30
			491.3	11	18
			1151.6	45	54
			1094.5	45	54
YQEPVLGPVRGFPFIIV	940.920	80	882.4	33	24
			730.6	39	38
			441.3	41	24

Table-SI 3. Sequences of the primer pairs used for RT-qPCR.[13,37,38]

Gene	Direction	Sequence (5′ to 3′)	Amplicon length, bp
TAS2R1	Forward	AAATGGCTCCGCTGGATCTC	172
	Reverse	GTGGCAAGCCAAAGTTCCAA	
TAS2R3	Forward	GGGACTCACCGAGGGGGTGT	160
	Reverse	CCTCAAGAGTGCCAGGGTGGTG	
TAS2R4	Forward	GCAGTGTCTGGTTTGTGACC	168
	Reverse	GCGTGATGTACAGGCAAGTG	
TAS2R5	Forward	ACACTCATGGCAGCCTATCC	107
	Reverse	CGAGCACACACTGTCTTCCA	
TAS2R7	Forward	GCAGGTGTGGATGTCAAACTC	167
	Reverse	TCTTGACCCAGTCCATGCAG	
TAS2R8	Forward	ATGTGGATTACCACCTGCCT	135
	Reverse	GGAAATGGCAAAGCATCCCAG	
TAS2R9	Forward	GCAGATTCGACTGCATGCTAC	70
	Reverse	TGCCTTTATGGCCCTCATGT	
TAS2R10	Forward	GCTACGTGTAGTGGAAGGCA	73
	Reverse	TCCATTCCCCAAAACCCCAA	
TAS2R13	Forward	GAAAGTGCCCTGCCGAGTAT	177
	Reverse	CCAGATCAGCCCAATTCTGGA	
TAS2R14	Forward	CCAGGTGATGGGAATGGCTTA	128
	Reverse	AGGGCTCCCCATCTTTGAAC	
TAS2R16	Forward	ATGGCATCACTGACCAAGCA	255
	Reverse	TTTCAACGTAGGGCTGCTCA	
TAS2R19	Forward	TCTTAGGACACAGCAGAGCA	146
	Reverse	AGCGTGTCATCTGCCACAAAA	
TAS2R20	Forward	ATTTGGGGGAACAAGACGCT	183
	Reverse	ACTACGGAAAAACTTGTGGGAA	
TAS2R30	Forward	GGCTGGAAAAGCAACCTGTC	191
	Reverse	ACACAATGCCCCTCTTGTGA	
TAS2R31	Forward	TTGAGGAGTGCAGTGTACCTTTC	218
	Reverse	ACGGCACATAACAAGAGGAAAA	
TAS2R38	Forward	CCCAGCCTGGAGGCCCACATT	216
	Reverse	TCACAGCTCTCCTCAACTTGGCA	
TAS2R39	Forward	TTCTGTGGCTGTCCGTGTTTA	207
	Reverse	GGGTGGCTGTCAGGATGAAC	
TAS2R40	Forward	CGGTGAACACAGATGCCACAGATA	150
	Reverse	GTGTTTTGCCCCTGGCCCACT	
TAS2R41	Forward	GCAGCGAATGGCTTCATTGT	223
	Reverse	TGGCTGAGTTCAGGAAGTGC	
TAS2R42	Forward	TCCTCACCTGCTTGGCTATC	161
	Reverse	GGCAAGCCAGGTTGTCAAGT	
TAS2R43	Forward	ATATCTGGGCAGTGATCAACC	148
	Reverse	CCCAACAACATCACCAGAATGAC	
TAS2R45	Forward	AGTACCCTTTACTGTAACCC	170
	Reverse	AGTAAATGGCACGTAACAAG	

**Gene**	**Direction**	**Sequence (5′ to 3′)**	**Amplicon length, bp**
TAS2R46	Forward	ACATGACTTGGAAGATCAAACTGAG	200
	Reverse	AGCTTTTATGTGGACCTTCATGC	
TAS2R50	Forward	CGCAAGATCTCAGCACCAAGGTC	151
	Reverse	GCCTTGCTAACCATGACAACCGGG	
TAS2R60	Forward	GGTGTTCAGTGCTGCAGGTA	156
	Reverse	CACCTTGAGGAACGACGACT	
GAPDH	Forward	AGGTCGGAGTCAACGGATTTG	94
	Reverse	GGGGTCATTGATGGCAACAATA	
PPIA	Forward	CCACCAGATCATTCCTTCTGTAGC	144
	Reverse	CTGCAATCCAGCTAGGCATGG	

Figures

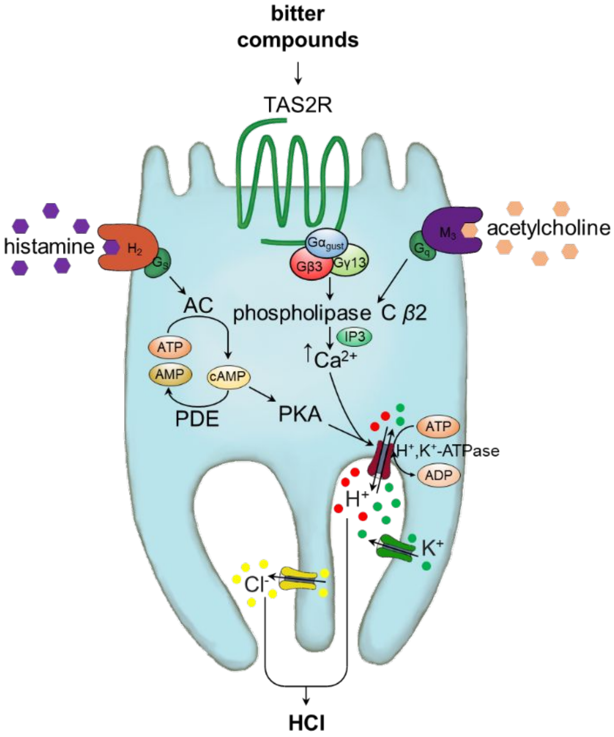

Figure-SI 1. Illustration of the proposed mechanism of proton secretion induced by bitter compounds in HGT-1 cells.[5,56]

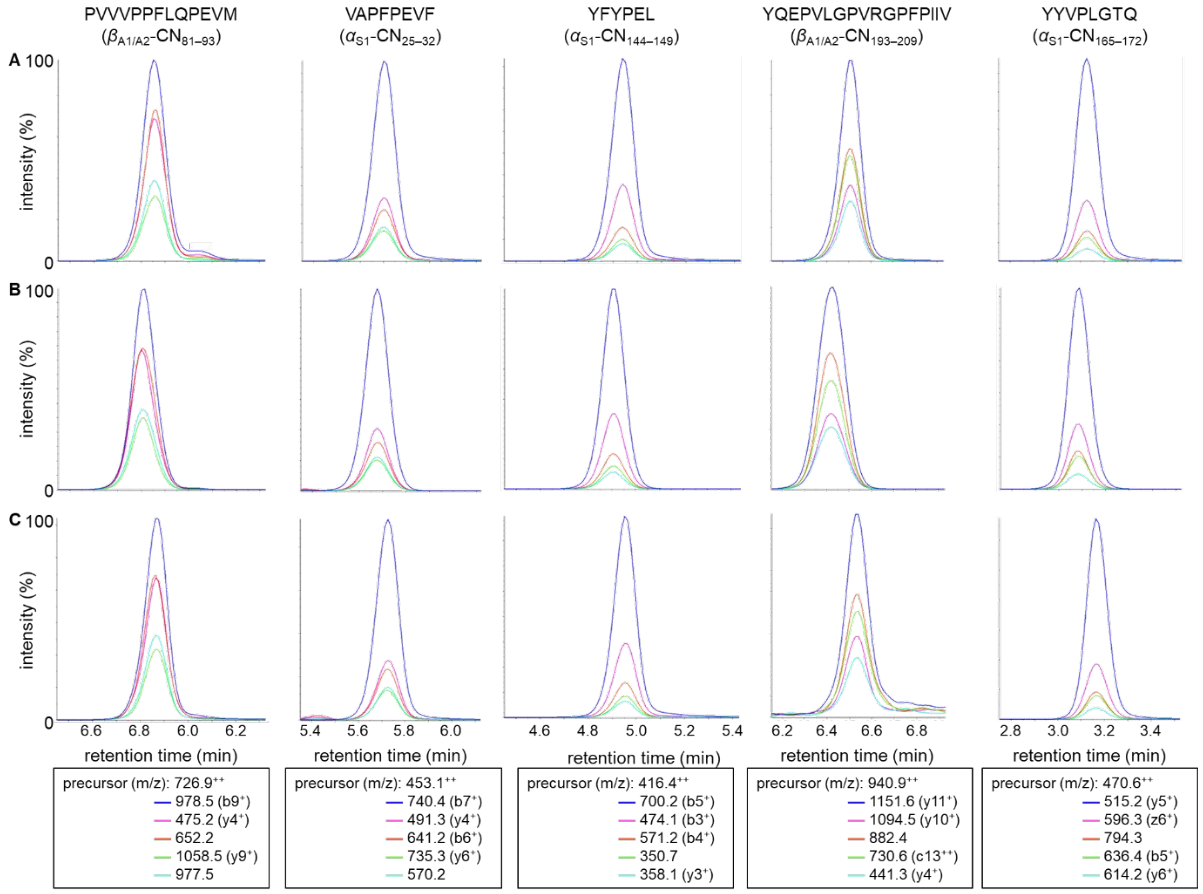

Figure-SI 2. Comparison of retention times (UPLC) and SRM mass transitions (MS/MS; 5 transitions per peptide; transitions identified with skyline 21.1.0.146) of PVVVPPFLQPEVM, VAPFPEVF, YFYPEL, YQEPVLGPVRGPFPIIV and YYVPLGTQ as (A) synthesized standard, (B) formed in *in vitro* digestion, and (C) formed in *in vivo* digestion.

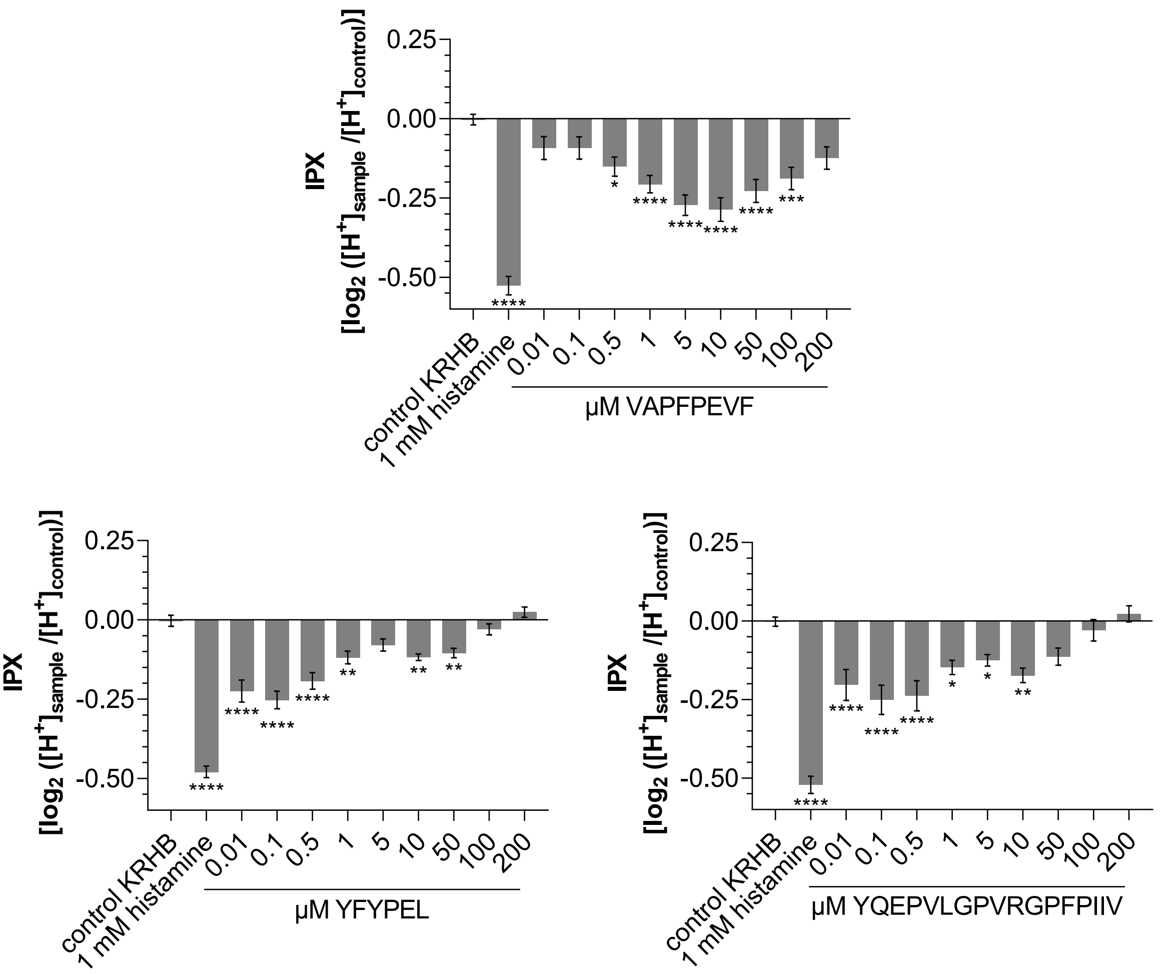

Figure-SI 3. Effect on proton secretion of HGT-1 cells incubated with VAPFPEVF (top) and the peptides YFYPEL and YQEPVLGPVRGPFPIIV with similar IPX profile (bottom). Data shown as mean ± SEM after incubation for 10 minutes, n = 4 – 8, t. r. = 4 – 6, control: KRHB, Statistics: one-way ANOVA Holm-Šidák post hoc test; significant differences are expressed with * = $p \leq 0.05$, ** = $p \leq 0.01$, *** = $p \leq 0.001$, **** = $p \leq 0.0001$.

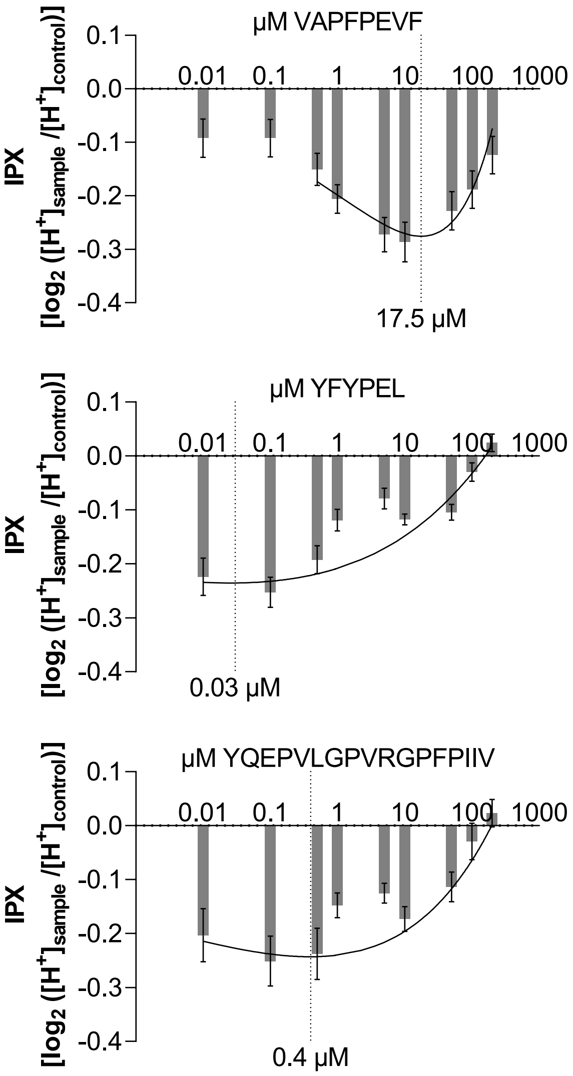

Figure-SI 4. Curve fit calculations of the proton secretion profile of the three peptides to determine which concentration has the greatest effect on proton secretion activity in HGT-1 cells in each case.

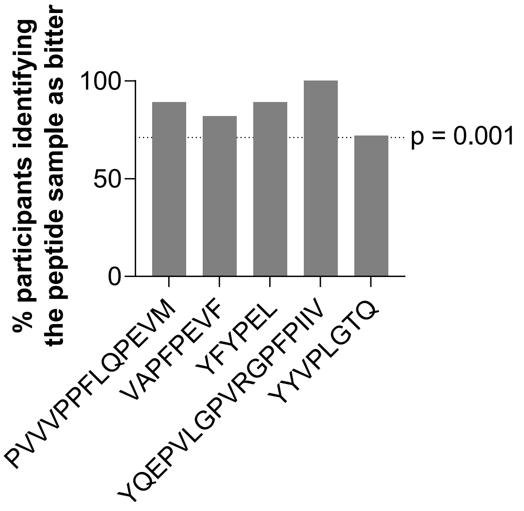

Figure-SI 5. Sensory experiments revealed significant bitterness ($p \leq 0.001$) toward water for all five selected peptides.

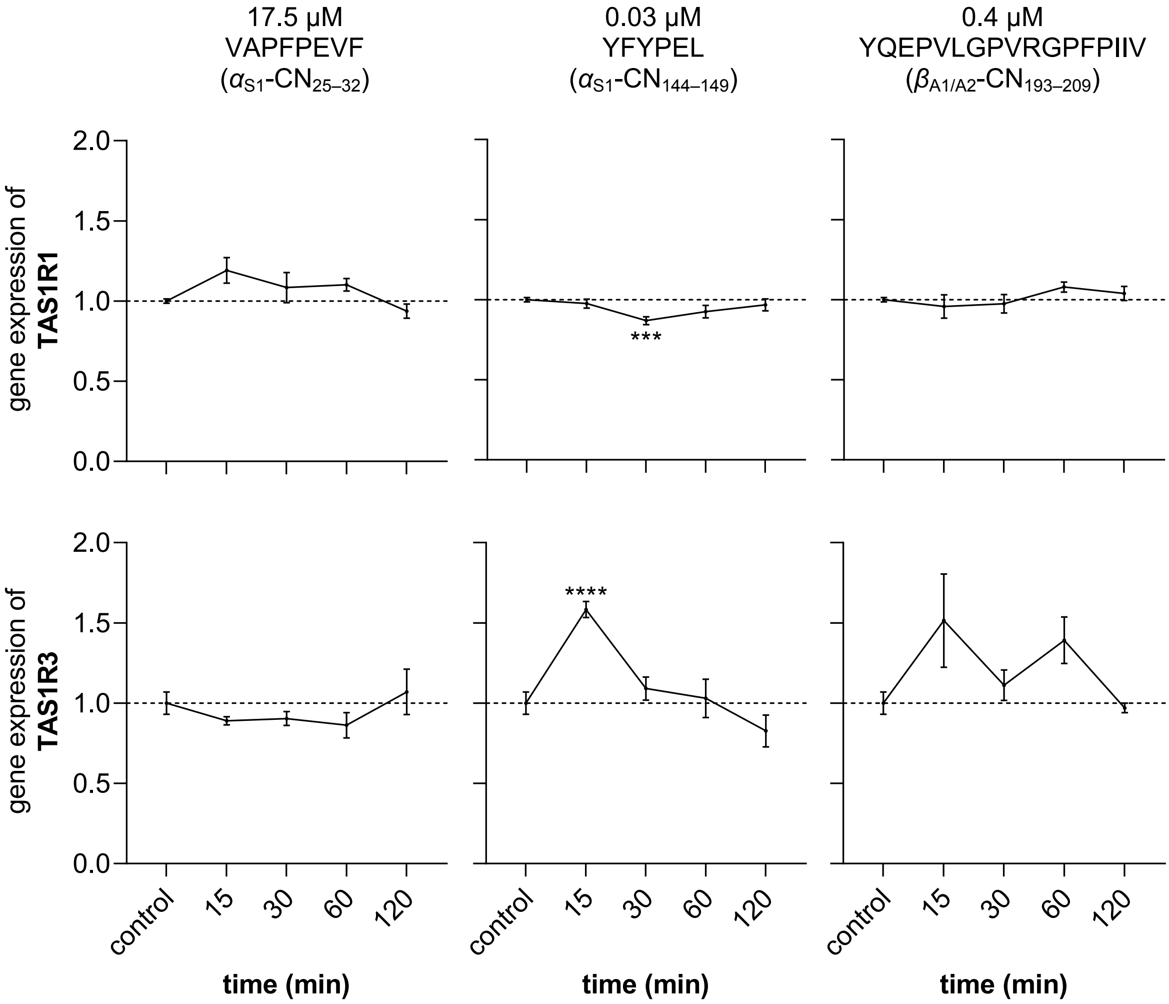

Figure-SI 6. Changes in gene expression (fold change) of taste receptors TAS1R1 (top) and TAS1R3 (bottom) as a function of incubation time with peptides VAPFPEVF (17.5 µM; left), YFYPEL (0.03 µM; center) and YQEPVLGPVRGPFPIIV (0.4 µM; right). Normalized to the expression of PPIA and GAPDH (reference genes). Data shown as mean ± SEM, n = 3, t. r. = 3, Statistics: t test Holm-Šidák method; significant differences are expressed with *** = $p \leq 0.001$, **** = $p \leq 0.0001$.

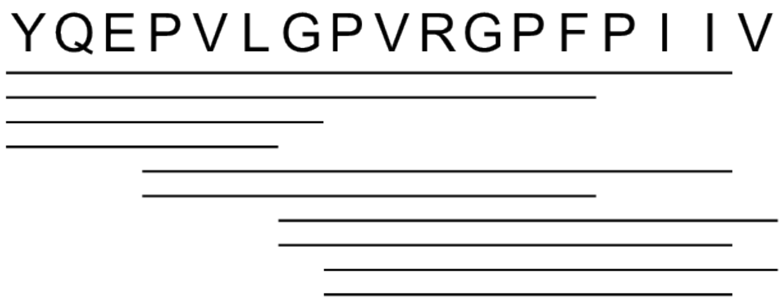

Figure-SI 7. Identified degradation products of YQEPVLGPVRGPFPIIV ($\beta_{A1/A2}$-$CN_{193–209}$) found in the further course of digestion explaining the degradation of the peptide.

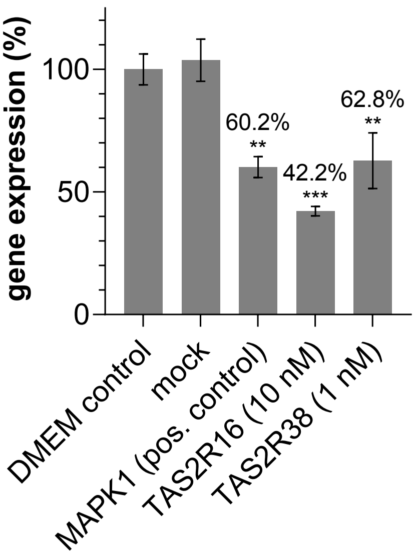

Figure-SI 8. Gene expression was reduced by transient transfection (knock-down) after 72 h. Mock transfections were performed with siRNA without target. Reduction in expression was best achieved with 10 nM siRNA targeting TAS2R16 (HSS121396) and 1 nM siRNA targeting TAS2R38 (HSS108754) to 42.2 and 62.8%, respectively.

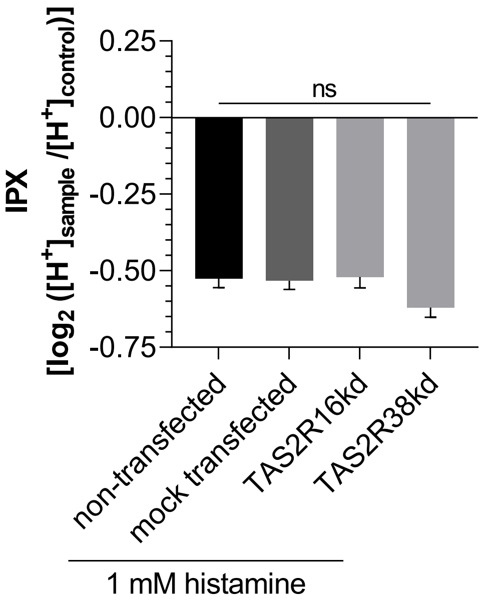

Figure-SI 9. TAS2R-independent histamine-induced stimulation of proton secretion does not differ from non-transfected cells in either mock-transfected or TAS2R16 or TAS2R38 knockdown HGT-1 cells.
